# Cathepsin C–Catalyzed Ligation Generates Intralysosomal Amyloid Fibrils from Dipeptide Esters

**DOI:** 10.64898/2025.12.23.696283

**Published:** 2025-12-25

**Authors:** Ruben D. Elias, Robert T. O’Neill, Idil I. Demiralp, Seth Allen, Daniel Serwas, Hannah Siems, Elizabeth A. Montabana, Carl Ash, Daniella A. Yacoubian, Oren L. Lederberg, Benjamin F. Cravatt, David A. Agard, Jeffery W. Kelly

**Affiliations:** 1 Department of Chemistry, The Scripps Research Institute; La Jolla, CA, USA; 2 Biohub, San Francisco; Redwood City, CA, USA; 3 Department of Biochemistry & Biophysics, University of California; San Francisco, CA, USA

## Abstract

Amyloid fibril-associated endolysosomal dysfunction is implicated in multiple neurodegenerative diseases. We report the rapid generation of intralysosomal amyloid fibrils by simply treating cells with certain dipeptide methyl esters. Cathepsin C mediates the ligation of dipeptides into oligopeptides that, sequence-dependently, self-assemble into amyloid fibrils. Progressive fibril growth, not fibril deposition, mediates lysosomal membrane permeabilization. Cryo-electron tomography studies reveal intralysosomal fibrils and broken lysosomal membranes upon dipeptide treatment. Certain oligopeptide fibril structures are competent to cross-seed the aggregation of neurodegeneration-associated Tau(P301S) at lysosomal sites. Similarly, the degree of lysosomal membrane permeabilization and ESCRT-repair response can be tuned with dipeptide sequence variation. The presented Cathepsin C-dependent amyloid fibril formation approach lends itself toward the development of novel tools to further probe lysosomal biology and pathobiology.

The causal relationship between autolysosome dysfunction and the onset and progression of neurodegenerative diseases has become a major research focus (1–6). A prion-like transmission of amyloid burden from affected to unaffected cells is proposed to drive pathology of certain amyloidoses, including tauopathies and synucleopathies (7, 8), where endocytosis of extracellular amyloid seeds, followed by their eventual endolysosomal breakout, mediates seeding (9–13). Accordingly, autolysosomal deacidification and markers for lysosomal membrane damage are observed in murine and neuronal models of Alzheimer’s disease (14, 15). Endolysosomal amyloid fibril breakout is thought to be mediated by the membranolytic properties of amyloid assemblies (16). Yet, cellular models of this endolysosomal breakout are imprecise, consisting of supplementing cell culture medium with preformed amyloid fibrils and exposing cells for extended periods (typically 1–2 days) before observing a given effect (9, 12, 17), resulting in heterogeneity in the number of affected cells and in subcellular localization of the endocytosed amyloid at a given timepoint.

L-leucyl-L-leucine methyl ester (LLOMe, Fig. 1A) has emerged as a routine biochemical reagent to induce mild to acute lysosomal membrane damage upon addition to cell culture media, facilitating the investigation of cellular lysosomal repair mechanisms (18–25). The mechanism of action of LLOMe has previously been characterized to involve its ligation into membranolytic (Leu-Leu)_n_-OH and (Leu-Leu)_n_-OMe oligopeptides (hereafter designated (Leu-Leu)_n_, where n=2–3 under previous experimental conditions (26)). (Leu-Leu)_n_ oligomer formation was identified to be catalyzed by the incompletely characterized ligase activity of the lysosomal dipeptidase cathepsin C (CTSC)(27–29). Despite widespread use of LLOMe as a lysosome damaging agent, the mechanistic basis of (Leu-Leu)_n_ membranolytic function has historically been unclear.

## (Leu-Leu)_n_ oligopeptides self-assemble into cross β-sheet amyloid fibrils

**Fig. 1.**
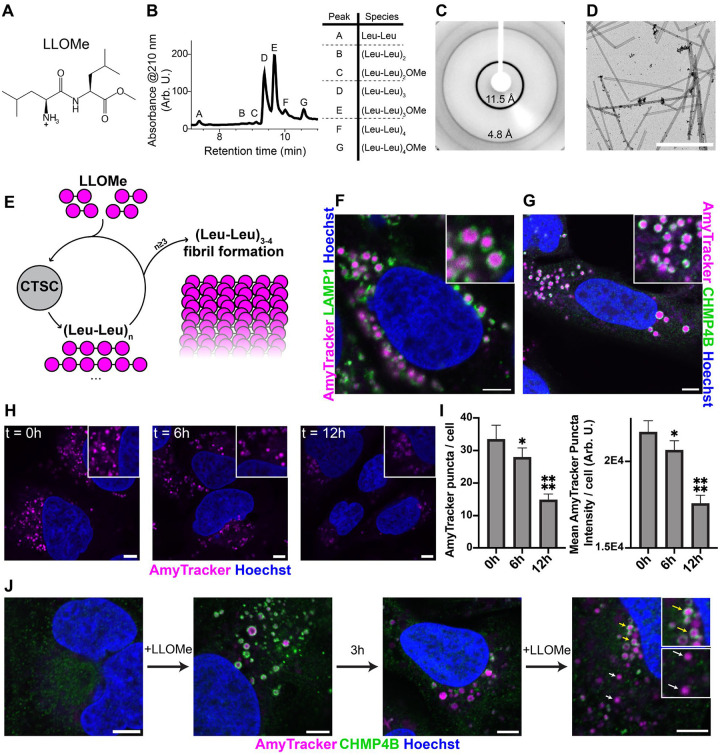
LLOMe is converted into (Leu-Leu)_n_ oligopeptides that spontaneously form β-sheet rich amyloids within endolysosomes. (**A**) Structure of LLOMe. (**B**) LC-MS chromatogram of LLOMe (100 mM) + CTSC (500 nM) overnight reaction precipitate dissolved in DMSO (left); assigned peaks identified by mass (right). (**C**) X-ray diffraction pattern of LLOMe + CTSC reaction precipitate. (**D**) Negative-stain electron microscopy of LLOMe + CTSC reaction mixture. (**E**) Schematic of CTSC-mediated LLOMe ligation and consequent fibril formation. (**F**) Representative image of U-2 OS cells treated with LLOMe (1 mM, 10 minutes) fixed and stained against endolysosomal marker LAMP1 and AmyTracker680 (2 μg/mL). (**G**) Representative image of CHMP4B association about AmyTracker puncta after LLOMe exposure (1 mM, 10 minutes). (**H**) Representative images and (**I**) quantitation of AmyTracker puncta number and intensity of U-2 OS cells treated with LLOMe (1 mM, 10 minutes) followed by LLOMe washout and further incubation for the indicated amount of time; n≥149 cells per timepoint, standard error of the mean shown as error bars, comparisons between the denoted and previous timepoint analyzed using one-way ANOVA, ****=P≤0.0001, *=P<0.05. (**J**) Representative images showing CHMP4B and AmyTracker staining before and after LLOMe exposure (1 mM, 10 minutes), 3 hours after LLOMe washout, and after a second LLOMe exposure (1 mM, 10 minutes). Yellow arrows distinguish AmyTracker puncta surrounded by CHMP4B. All scalebars=5 μm.

Due to their high hydrophobicity, low solubility, and high β-strand propensity, we hypothesized that (Leu-Leu)_n_ oligopeptides would spontaneously self-assemble into amyloid fibrils. Combining CTSC and LLOMe in phosphate buffer (pH 6.5) resulted in the formation of a precipitate. Liquid chromatography–mass spectrometry (LC-MS) analysis of the solubilized precipitate revealed the presence of (Leu-Leu)_1–4_ oligopeptides, primarily (Leu-Leu)_3_ (Fig. 1B), in agreement with a previous study (26). To probe structural order within the precipitate, we conducted an X-ray diffraction study, revealing diffraction rings at 11.5 and 4.8 Å (Fig. 1C), hallmarks of a cross-β-sheet amyloid fibril structure (30). Additionally, negative stain electron microscopy revealed rod-like assemblies in the reaction mixture (Fig. 1D). Collectively, these results suggest that CTSC-catalyzed ligation of LLOMe affords (Leu-Leu)_3–4_ oligopeptides, that, when they exceed their critical concentration, rapidly self-assemble to form new or add onto existing amyloid fibrils (Fig. 1E).

In U-2 OS cells, LLOMe treatment resulted in visibly expanded LAMP1-positive endolysosomes (Fig. S1A), possibly due to the genesis of lumenal amyloid fibrils. To probe amyloid content within endolysosomes, cells were stained with the amyloid-binding dye AmyTracker (31), resulting in robust staining within endolysosomes (Fig. 1F). We observed similar results using amyloid-binding fluorophores thioflavin T and Proteostat (Fig. S1B, C). Additionally, the nuclear stain Hoechst 33442, characterized as amyloid-sensitive (32, 33), faintly stained extranuclear puncta colocalizing with AmyTracker and Proteostat (Fig. S1D, E). The AmyTracker signal was not derived from LLOMe-induced lysosomal deacidification, as lysosome deacidification using bafilomycin A1 alone yielded no AmyTracker puncta (Fig S1F). Pretreatment with the CTSC inhibitor AZD5248, which forms a covalent bond with the active site Cys234 residue (34), ablated LLOMe-induced AmyTracker staining and endolysosomal expansion (Fig. S1G), confirming CTSC activity is responsible for the generation of amyloid fibrils. AmyTracker puncta were predominantly encircled by CHMP4B, a marker for ESCRT-mediated lysosomal repair (Fig. 1G)(18, 19), suggesting that amyloid formation causes lysosomal damage. Overall, we provide *in vitro* and in-cell evidence for the spontaneous amyloid fibril formation of CTSC-derived (Leu-Leu)_n_ oligopeptides, and that these intralysosomal amyloid fibrils mediate lysosomal damage.

## After amyloidogenesis, remnant (Leu-Leu)_n_ amyloid fibrils are membranolytically inert

LLOMe-induced lysosomal damage is commonly carried out in a ‘reversible’ manner by removing LLOMe after a given treatment window (19, 35). Accordingly, we hypothesized that (Leu-Leu)_n_ amyloid fibrils would be short lived. In agreement with previous results (18), we observed that after brief LLOMe treatment, CHMP4B was rapidly recruited to endolysosomes and dissipated within 180 minutes after LLOMe washout (Fig. S2); however endolysosomes remained swollen at this timepoint. Strikingly, 6 hours after LLOMe washout, cells were still robustly amyloid laden (AmyTracker), with puncta dimly present even after 12 hours (Fig. 1H, I). This suggests that the membranolytic activity of (Leu-Leu)_n_ amyloid fibrils only occurs during active fibril growth (amyloidogenesis), and that after LLOMe washout, previously formed (Leu-Leu)_n_ amyloid fibrils are membranolytically inert and are cleared within endolysosomes over tens of hours. Scrutinizing this hypothesis, we treated cells with LLOMe followed by washout, waited three hours for ESCRT-mediated repair and machinery dissociation, then repeated LLOMe treatment. After the second LLOMe treatment we observed two sets of AmyTracker puncta: one surrounded by CHMP4B rings and one not (Fig. 1J), suggesting the presence of both membranolytic and inert (Leu-Leu)_n_ amyloid fibril deposits. Accordingly, we speculate that (Leu-Leu)_n_ amyloid fibril membranolytic activity arises from physical interactions between growing amyloid fibrils and the endolysosomal membrane (16).

## Cryo-electron tomography reveals fibril-laden, damaged lysosomes

**Fig. 2.**
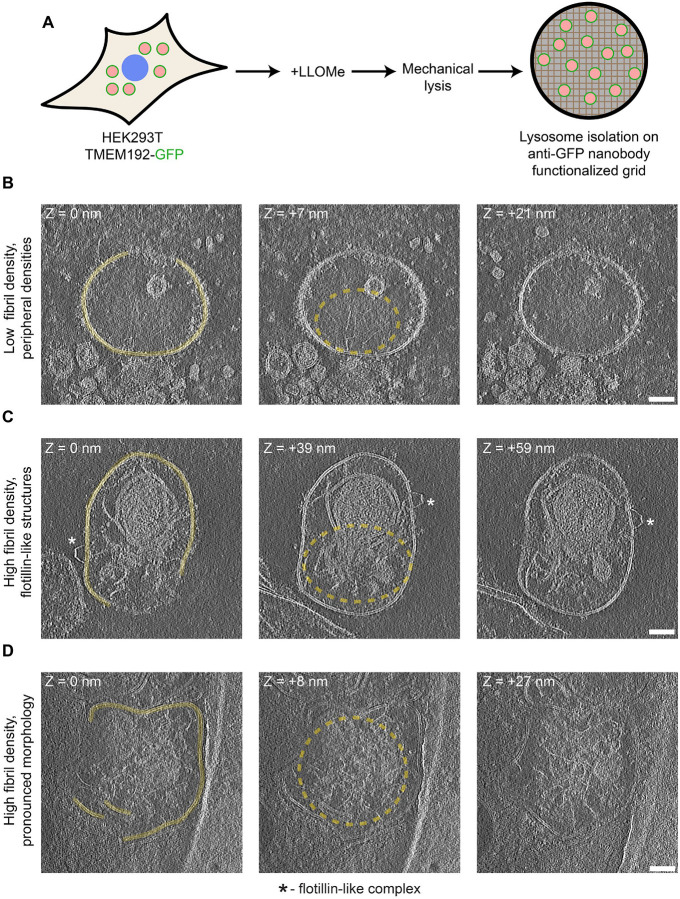
Cryo-electron tomography of isolated LLOMe-exposed lysosomes. (**A**) Schematic of on-grid affinity isolation of lysosomes from TMEM192-GFP-expressingHEK293T cells treated with LLOMe (1 mM, 10 minutes). (**B-D**) Representative 2D tomogram slices of isolated lysosomes with (**B**) low lumenal fibril density with peripheral, ‘fuzzy’ densities, (**C**) high lumenal fibril density with peripheral flotillin-like complexes, and (**D**) high lumenal fibril density with pronounced, misshapen membrane morphology. (pixel size=1.51 Å, scalebar=50 nm). Z-axis distance between slices relative to the first slice in each series is denoted. Continuous membranes are highlighted, a dashed circle highlights fibril density, and * denotes flotillin-like structures.

To provide additional evidence for (Leu-Leu)_n_ intralysosomal amyloid fibril formation and deleterious membrane interactions, we conducted cryo-electron tomography studies employing a strategy to affinity isolate lysosomes. HEK293T cells stably expressing the lysosomal membrane protein TMEM192-GFP were treated with LLOMe, lysed using mechanical disruption, and the lysate containing intact lysosomes was incubated on anti-GFP nanobody-functionalized grids before plunge freezing and tilt series collection (Fig. 2A). Using this strategy, we observed lumenal, fibrous cargo with observable fibril-membrane contacts in a subset of observed lysosomes (Fig. 2B–D). In many cases we additionally observed the accumulation of membrane associated and membrane-spanning ‘fuzzy’ densities (Fig. 2B), likely indicative of the recruitment of lysosomal repair machineries. We furthermore rarely observed flotillin-like complexes on the membrane surface (Fig. 2C), in agreement with previous studies (36, 37). Lysosomal membranes were often discontinuous along a given region, with prominently misshapen membranes occurring concurrently with high lumenal fibril densities (Fig. 2 D). Overall these observations provide visible evidence that LLOMe treatment of cells results in lysosomal membrane disruption linked with the accumulation of lumenal amyloid fibrils.

## LLOMe induces the aggregation of cytosolic Tau(P301S)

**Fig. 3.**
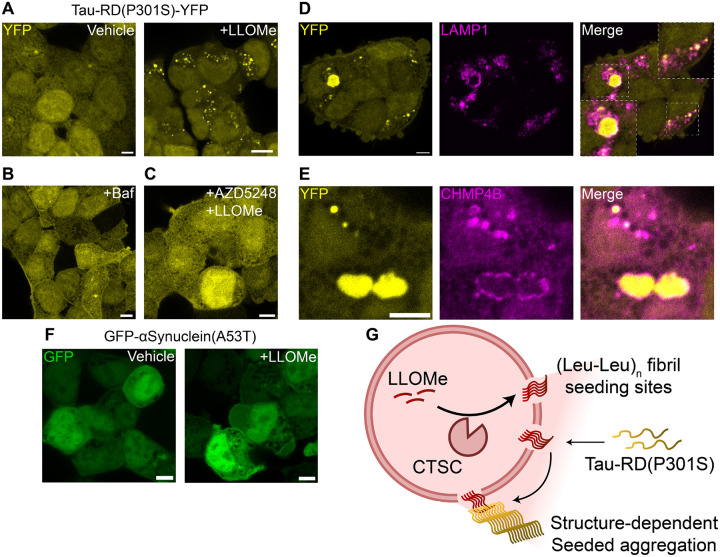
(Leu-Leu)_n_ amyloid fibrils seed the aggregation of cytosolic aggregation-prone Tau. (**A-C**) Representative images of HEK293T cells expressing Tau-RD(P301S)-YFP showing (**A**) puncta formation upon LLOMe exposure (1 mM, 60 minutes) or lack of puncta formation upon (**B**) bafilomycin A1 treatment (250 nM, 180 minutes) or (**C**) AZD5248 pretreatment (10 μM, 90 minutes) before LLOMe exposure (1 mM, 60 minutes). (**D-E**) Representative images of colocalization of small Tau-RD(P301S) puncta and encirclement of large aggregates by (**D**) LAMP1 or (**E**) CHMP4B. (**F**) Representative images of GFP-αSynuclein(A53T) before and after LLOMe exposure (1 mM, 60 minutes). (**G**) Schematic of proposed mechanism by which (Leu-Leu)_n_ amyloids seed the aggregation of certain susceptible cytosolic amyloidogenic proteins. All scalebars=5 μm.

A recent report indicated that endocytosed intralysosomal tau fibrils can seed aggregation of cytosolic tau at the lysosomal membrane-cytosol interface (10). Given that we observed intralysosomal fibril-membrane disruptions, we hypothesized that (Leu-Leu)_n_ amyloid fibrils could behave similarly to cross-seed the aggregation of Tau. Utilizing overexpression of cytosolic Tau-RD(P301S)-YFP as a reporter in HEK293T cells, we observed the formation of bright puncta within one hour of LLOMe exposure (Fig. 3A). Tau-RD(P301S) puncta formation was not observed upon treatment with bafilomycin A1 or cotreatment of LLOMe with CTSC inhibitor AZD5248 (Fig. 3B, C), confirming that Tau-RD(P301S) aggregation was not the result of lysosomal deacidification, but instead required the LLOMe-CTSC interaction. Puncta were either surrounded by or overlayed with endolysosomal marker LAMP1 (Fig. 3D) or CHMP4B (Fig. 3E), suggesting Tau-RD(P301S) aggregation occurred at sites of lysosomal damage. We next examined whether LLOMe could cross-seed another amyloidogenic reporter, GFP-αSynuclein(A53T). However, αSynuclein(A53T) remained dispersed upon LLOMe exposure (Fig. 3F), suggesting the (Leu-Leu)_n_ amyloid structural match was lacking for αSynuclein seeding. Altogether we evidence that (Leu-Leu)_n_ amyloid fibrils produced upon LLOMe exposure harbor relevant structure to seed the aggregation of Tau-RD(P301S) (Fig. 3G).

## Varying dipeptide composition tunes the CTSC-mediated endolysosomal damage response

**Fig. 4.**
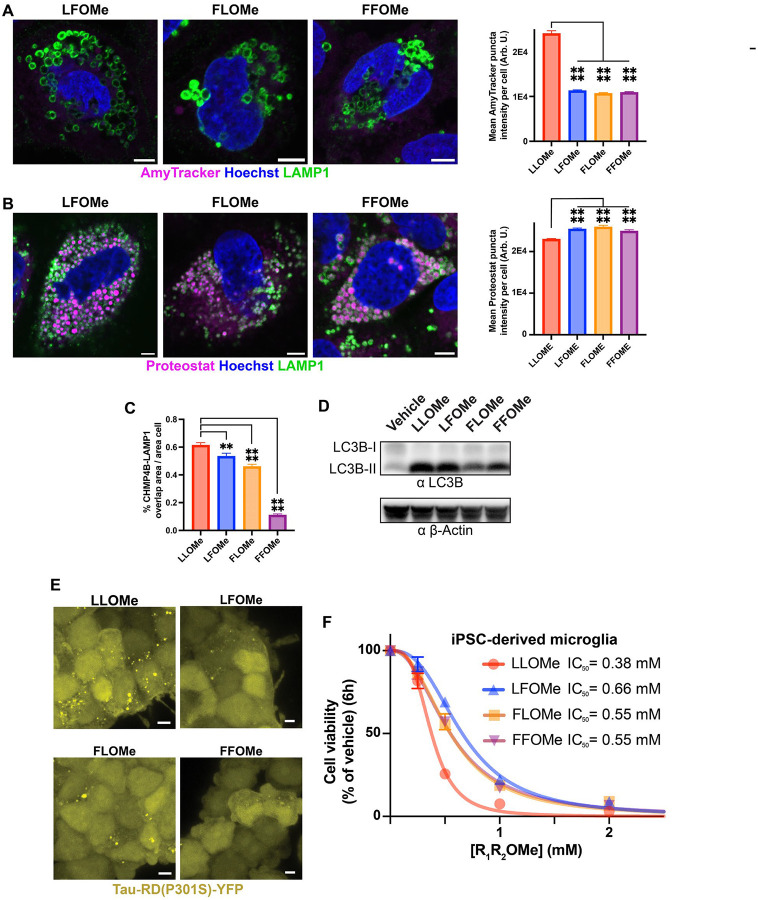
Implied structural heterogeneity of phenylalanine-containing dipeptide methyl esters imparts different phenotypes than LLOMe. (**A-B**) Representative images of U-2 OS cells treated with the indicated dipeptide methyl esters (1 mM, 60 minutes) stained for LAMP 1 and (**A**) AmyTracker or (**B**) Proteostat, with accompanying plots displaying mean puncta fluorescence intensity of the corresponding dye (n≥135 cells in (**A**) or n≥522 cells in (**B**), standard error of the mean shown as error bars), (**C**) Percent CHMP4B-LAMP1 overlap are per cell area (n≥242 cells per treatment, standard error of the mean shown as error bars); indicated comparisons analyzed using one-way ANOVA, ****=P≤0.0001, **=P<0.01. (**D**) Western blot displaying accumulation of LC3B-II upon treatment of the indicated dipeptide methyl esters (1 mM, 60 minutes). (**E**) Representative images of Tau-RD(P301S)-YFP puncta formation in HEK293T upon treatment of indicated dipeptide methyl esters (1 mM, 60 minutes). (**F**) Plot of viability of iPSC-derived microglial cultures against 6-hour dipeptide methyl ester exposure of the indicated concentration; n=3 wells per datapoint, standard deviation shown as error bars where possible, data fit to variable slope IC_50_ curves. All scalebars=5 μm.

We anticipated that further exploration of the CTSC-dipeptide ligation platform through dipeptide sequence variation could reveal distinct aggregate/amyloid structures and cellular phenotypes. Seeking to design dipeptides with similar properties to LLOMe, we chose to incorporate phenylalanine, an aromatic, hydrophobic residue that is itself amyloidogenic (38). The dipeptides LFOMe, FLOMe, and FFOMe were readily ligated into tetrapeptides by CTSC-mediated ligation *in vitro* (Fig. S3A–C). Unlike LLOMe, cell treatment with these dipeptides led to no or very poor AmyTracker fluorescence (Fig. 4A, vehicle and LLOMe examples in Fig. S4A); however, endolysosomal vesicles were visibly enlarged by all dipeptide-OMe treatments (Fig. S4B), suggestive of the intralysosomal buildup of oligopeptide products/aggregates. Proteostat staining within endolysosomal vesicles was robust (Fig. 4B, vehicle and LLOMe examples in Fig. S4C), arguing that LFOMe-, FLOMe-, and FFOMe-derived oligopeptide aggregates may differ structurally from LLOMe-derived cross-β-sheet structures. Recruitment of CHMP4B to endolysosomes was diminished using LFOMe and FLOMe relative to LLOMe; however, FFOMe invoked little to no CHMP4B puncta localization to endolysosomes (Fig. 4C, Fig. S4D). In contrast, we observed LC3B-II accumulation, a marker of lysophagy (22) and ATG8-mediated lysosomal damage response (23, 39), with all dipeptides studied (Fig. 4D). LFOMe and FLOMe weakly induced Tau-RD(P301S) puncta formation in HEK293T cells, while FFOMe did not detectably do so (Fig. 4E). We posit that FFOMe is a lysosomal damaging agent that negligibly invokes the ESCRT repair response relative to LLOMe, apparently through the CTSC-mediated formation of oligomers that form predominantly non-amyloid aggregates that are incapable of Tau-RD(P301S)-seeding.

Lysosomal damage-mediated cell death (40) was characterized utilizing LLOMe, LFOMe, FLOMe, and FFOMe in iPSC-derived microglia, which exhibit high lysosomal function (41, 42) and are likely to be more susceptible to lysosomal damage-mediated cell death than HEK293T or U-2 OS cells (LLOMe was historically used to kill immune cells (26)). Consistent with this, a major loss in viability was observed after only a 6-hour exposure of 0.5 mM LLOMe, resulting in an IC_50_ of 0.38 mM (Fig. 4F). LFOMe, FLOMe, and FFOMe displayed slightly reduced toxicities, with IC_50_’s of 0.66, 0.55, and 0.55 mM respectively, suggesting a link between microglial cell death and dipeptide-derived aggregate structures. Further exploration into the relationship between sequence-derived aggregate structure, resulting lysosomal damage, and microglial and neuronal death can further inform the roles of intralysosomal aggregates in neurodegenerative diseases.

## A reactive CTSC-dipeptide thioester intermediate mediates dipeptide ligation

We next investigated the peptide structural requirements for the CTSC-mediated amyloid fibril generation. Neither Leu-Leu (LLOH), LLOMe diastereomers at either residue, nor N,N-dimethyl LLOMe formed AmyTracker puncta or visibly expanded endolysosomes upon cell treatment (Fig. S5A), implying the necessity of the methyl ester, L stereochemistry, and an NH_3_^+^ termini–CTSC(Asp1) interaction unobstructed by additional methyl groups, an interpretation supported by a molecular docking study (Fig. S5B) (43). To probe whether CTSC-mediated ligation is restricted to dipeptides, we employed LOMe, LLLOH, and LLLOMe in cells. Neither LOMe nor LLLOH resulted in the formation of AmyTracker puncta; however, LLLOMe resulted in robust AmyTracker puncta formation (Fig. S5C), implicating the combination of the methyl ester and a Leu-based peptide chain ≥2 as necessary for productive CTSC-mediated ligation and subsequent amyloid fibril formation.

Delving further into the substrate requirements of the CTSC peptide ligation reaction, we performed three *in vitro* reactions with CTSC: first, using LOMe, second, using LOMe + LLOMe, and third, using the tripeptide GLLOMe (used in place of LLLOMe due to increased solubility), and characterized reaction product masses using LC-MS. LOMe alone did not react with CTSC (Fig. S6A); however, addition of equimolar amounts of LLOMe and LOMe resulted in the formation of L-LLOMe and L-LL-LLOMe (Fig. S6B). Intriguingly, GLLOMe + CTSC resulted in modest formation of LOMe and GL-GLLOMe (Fig. S6C), implying the hydrolysis of GL-LOMe to release LOMe, and ligation of GL- to the N-terminus of a new GLLOMe molecule. In light of this and previous results using LLLOH and LLLOMe, we hypothesize that the methyl ester drives the lysosomal accumulation of these peptides through a mechanism to be determined.

**Fig. 5.**
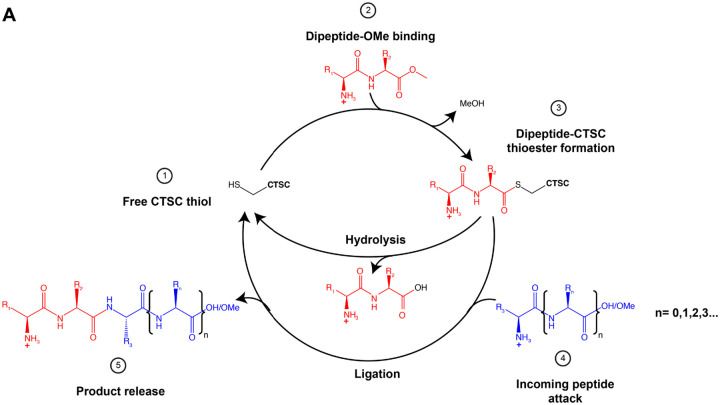
Proposed mechanism of CTSC-mediated dipeptide ligation. (**A**) Schematic of proposed mechanism of CTSC R_1_-R_2_-OMe ligation, where R_1_ and R_2_ belong to the set of natural and unnatural amino acids for which CTSC bears affinity. The formation of a thioester intermediate (3) can resolve through hydrolysis, releasing the dipeptide carboxylic acid, or (4) undergo nucleophilic attack by the N-terminus of an incoming peptide of length n, harboring residues R_3_…R_n_, which may belong to a distinct set of amino acids from R_1_-R_2_. This attack affords the release of a new peptide (5) of length 2+(1+n), composed of R_1_-R_2_-R_3_…R_n_.

Altogether, these observations, and those from pretreatment with the CTSC inhibitor AZD5248 ablating AmyTracker staining, permit us to speculate on the nature of the CTSC-dipeptide methyl ester interaction (Fig. 5A). We propose the initial reaction proceeds similar to the characterized subtiligase reaction mechanism (44): the R_1_R_2_OMe C-terminal carbonyl is attacked by a CTSC active site thiolate, ultimately releasing methanol and forming a metastable, populated thioester intermediate CTSC-R_1_R_2_ (45) (note here the C-terminal methyl ester functions as a leaving group, and can be functionally replaced with β-naphthylamide and O-benzyl groups (46) or by a polypeptide chain as would be the case in a typical CTSC aminopeptidase reaction). Dependent on reaction conditions, the thioester can either be hydrolyzed to release R_1_R_2_-OH, or CTSC can facilitate the nucleophilic attack on the thioester by the amino terminus of a second peptide H_2_N-R_3_…R_n_ in a CTSC-catalyzed backreaction akin to native chemical ligation (47) to release the product R_1_R_2_-R_3_…R_n_. From this model, the concentration of the nucleophilic peptide is important to affect ligation, however the nature of the C-terminus of the nucleophilic peptide is inconsequential. Indeed for LLOMe we observe both carboxylic acid and methyl ester oligopeptides (Fig. 1B). Additionally, the permissible length of the attacking peptide appears unfixed, as in the case of LLOMe we observe up to (Leu-Leu)_4_ oligopeptides, implying a (Leu-Leu)_3_ nucleophilic peptide (Fig. 1B). Even a single amino acid methyl ester, i.e. LOMe can serve as the nucleophile (Fig. S6B). Results using the tripeptide GLLOMe suggest the product length follows the trend 2+n, where n is the length of the attacking peptide. We essentially argue that aminopeptidase activity generates the ligase-competent CTSC-dipeptide thioester intermediate: CTSC initially acts as an aminopeptidase, removing two amino acids from a peptide N-terminus, forming a reactive thioester with said dipeptide, then either releases the dipeptide by thioester hydrolysis or ligates the dipeptide onto an exogenously supplied amino terminus-bearing amino acid/peptide present at a high local concentration.

## Discussion

To our knowledge, LLOMe is the first characterized biochemical reagent that, upon addition to cell culture media, induces the rapid intra-endolysosomal genesis of amyloid fibrils, a discovery that recontextualizes numerous previous studies carried out with this endolysosome damaging reagent. We present here a characterization of the rate of intralysosomal amyloid fibril clearance in U-2 OS cells (Fig. 1H, I), which demonstrates that endolysosomal clearance of amyloid fibrils is generally slow, despite taking place in an acidic, denaturing, and protease-rich environment. Indeed, in earlier investigations (48, 49), fluorescent Aβ42 fibrils were fed to neuronal cell cultures and remained within endolysosomal compartments for weeks. The (Leu-Leu)_n_ amyloid fibril structure has fewer residues per monomer participating in the cross-β-sheet structure, likely enabling faster dissociation of individual peptides from the fibril, which is probably rate-limiting for endoproteolysis. We suspect (Leu-Leu)_n_ fibrils induce Tau aggregation through primary or secondary nucleation. Further investigation into the (Leu-Leu)_n_ fibril structure may inform the mechanistic requirements for Tau self- and cross-seeding in neurodegenerative contexts. We additionally highlight the potential to engineer novel dipeptides, tripeptides, and beyond, which may afford more complex oligopeptide structures through CTSC ligation to more specifically induce aggregation of specific disease-associated amyloidogenic proteins that (Leu-Leu)_n_ amyloid cannot, such as αSynuclein(A53T).

In summary, dipeptide methyl ester treatment represents a simple method to generate pathologically relevant amyloid fibrils and other aggregate structures directly within the lysosome, dependent on the endogenously expressed enzyme CTSC. The displayed modularity of this system presents a platform rife with opportunity to generate novel tools to explore lysosomal biology and, in particular, to study neurodegenerative pathobiology.

## Supplementary Material

Supplement 1

Supplement 2

Supplement 3

Supplement 4

Materials and Methods

Figs. S1 to S30

References (51–53)

Movies S1 to S3

## Data Availability

The full Cryo-electron tomography dataset collected here is available on the CZ CryoET Data Portal (*deposition_number TBA*). For a control comparison dataset see (https://cryoetdataportal.czscience.com/datasets/10444). Relevant data from image analysis is available upon request. LFOMe, and FLOMe materials are available from the Kelly lab under the terms of an MTA
