# Supplementary material for: Cathepsin C–Catalyzed Ligation Generates Intralysosomal Amyloid Fibrils from Dipeptide Esters
Source: bioRxiv. 2025 Dec 25:2025.12.23.696283. Preprint. [Version 1] doi: 10.64898/2025.12.23.696283 (PMC12767319; doi:10.64898/2025.12.23.696283)
Supplement: Supplement 1 [file media-1.pdf]

## Materials and Methods

Materials

Primary antibodies used were mouse anti-LAMP1 (Cell Signaling catalog no. 15665), rabbit anti-CHMP4B (Proteintech catalog no. 501728932), rat anti-Galectin-3 (BioLegend catalog no. 125402), rabbit anti-LC3B (Cell signaling catalog no. 2775), Mouse anti-Vinculin (Sigma-Aldrich catalog no. V9131), mouse anti-Cathepsin C (Santa Cruz Biotechnology catalog no. sc-74590), and rabbit anti-β-Actin (Cell Signaling catalog no. 4970). Secondary antibodies for imaging, goat anti-mouse Alexa Fluor 488, goat anti-rabbit Alexa Fluor 647, and goat anti-rat Alexa Fluor 555 were purchased from Thermo Fisher Scientific (catalog no. A11001, A21245, and A21434 respectively). Secondary Antibodies for immunoblots, goat anti-rabbit-HRP and goat anti-mouse-HRP, were purchased from Cell Signaling (catalog no. 7074 and 7076 respectively) and visualized using SuperSignal West Pico PLUS substrate (Thermo Scientific catalog no. 34580). AmyTracker 680 was purchased from Ebba Biotech AB, Proteostat aggresome detection kit was purchased from Enzo (catalog no. ENZ-51035-0025), and thioflavin T was purchased from Sigma-Aldrich (catalog no. 596200). CellTiter Glo assay reagents were purchased from Promega Corporation (catalog no. G9242). AZD5248 was purchased from R&D systems inc. (catalog no. 7130), E64d was purchased from Bio-Techne (catalog no. 4545), and bafilomycin A1 was purchased from RPI (catalog no. B40500).

In-vitro CTSC-peptide methyl ester reactions and liquid chromatography-mass spectrometry

For $(Leu\text{-}Leu)_n$ products, to produce large enough precipitate mass to separate from the reaction mixture, LLOMe solid was dissolved directly into 20 mM sodium phosphate pH 6.5, 250 mM NaCl at a concentration of 100 mM, and the reaction was initiated by addition of 500 nM recombinant mouse active CTSC (R&D Systems catalog no. 2336-CY). The reaction was incubated at 37° C overnight with shaking, and the resulting precipitate was pelleted by centrifugation and air dried overnight after aspiration of supernatant. For LC-MS analysis, pellets were then redissolved in DMSO.

For all other reactions, peptides (from DMSO stocks) and CTSC were mixed at the indicated concentrations under the reaction conditions described above, and an aliquot of the reaction mixture was analyzed by LC-MS.

LC-MS analysis was carried out on an Agilent 1260 Infinity II. Sample was loaded onto a C18 column (Agilent catalog no. 827700-902) and eluted using 2-95% vol/vol acetonitrile gradient supplemented with 0.1% vol/vol TFA.

X-ray diffraction

X-ray diffraction was carried out on a Bruker Microstar APEX II CCD diffractometer equipped with Cu Kα radiation (λ = 1.54178 Å). LLOMe + CTSC dried precipitate was mounted on a cryoloop and data was collected at room temperature. A single Phi 360° scan was collected with a sample-to-detector distance of 60 mm and an exposure time of 5 min.

Negative stain transmission electron microscopy (TEM)

Carbon-coated copper grids (400 mesh) were glow-discharged and 10 µL of LLOMe + CTSC reaction mixture was adsorbed for 2 minutes. Excess sample was wicked away and grids were negatively stained with 50 µL 2% uranyl formate for 2 minutes. Excess stain was wicked away and the grids were allowed to dry. Samples were analyzed at 80kV with a ThermoFisher Talos L120C transmission electron microscope and images were acquired with a CETA 16M CMOS camera.

Cell culture

U-2 OS and HEK293T cells stably expressing Tau RD(P301S)-YFP were acquired from ATCC (HTB-96, and CRL-3275 respectively). Cells were cultured in DMEM (Thermo Fisher Scientific catalog no. 11995073) supplemented with 10% vol/vol fetal bovine serum, 100 U/ml penicillin, and 100 µg/ml streptomycin, and were maintained at 37° C supplemented with 5% $CO_2$.
iPSC culture and microglial differentiation were carried out as previously reported (*50*).

Cryo-electron tomography: Sample preparation, plunge freezing, and data collection

HEK293T cells stably expressing a carboxy-terminal GFP-tagged TMEM192 were treated with 1 mM LLOMe for 10 min. Cell were lysed lysis using a hypotonic homogenization buffer (25 mM Tris-HCl pH 7.5, 50 mM sucrose, 0.2 mM EGTA, 0.5 mM $MgCl_2$), followed by application of shearing forces generated using a 23-G syringe. The resulting lysate was immediately mixed with a sucrose buffer on ice (2.5 M sucrose, 0.2 mM EGTA, 0.5 mM MgCl2). The nuclear fraction was separated by centrifugation (1,000xg, 10 minutes, 4ºC). Supernatants were kept at 4ºC before freezing. The TMEM192-GFP positive lysosomes from this lysate were then purified on-grid using electron-microscopy grids functionalized with anti-GFP nanobodies (*51, 52*).

Plunge freezing was done using a Leica GP2, a Whatman #1 blotting paper and liquid ethane at -180 °C as a cryogen. Lysate was added to the functionalized grids in a series of a total of six 6 µl drops which were blotted away after each addition. Grids where washed with 6 µl PBS and a final 6 µl drop of PBS was added. The functionalized grids were loaded into the chamber, which was set at 4 °C and 75% humidity. Back-side blotting was then done for 5-6 s, and the grid was plunged into liquid ethane and then stored in liquid nitrogen.

All tilt series were collected on one grid on a Krios G4 equipped with an X-FEG electron gun, a Falcon 4i direct electron detector, and the SelectrisX energy filter. The pixel size was set to 1.51 Å per pixel, and the total dose to 62.93 e− Å−2 linearly spread over 31 tilt images spanning a range of –45° to +45° in 3° increments. The software used for data collection was TFS Tomo 5. The movie frames were saved in the EER format.

Motion correction, tilt-series alignment and tomogram reconstruction were all performed using AreTomo3 v1.0.7 (*53*).

Cell viability

Microglia were seeded in a 96-well plate (10,000 cells/well). After 48 hours, cell culture media was replaced with media containing the indicated concentrations of dipeptide. Cell viability was then analyzed using Cell-Titer Glo 2.0 per manufacturer's instructions. Luminescence data was normalized relative to vehicle control, plotted and fit to a variable slope Hill equation using GraphPad Prism.

Immunostaining

Cells were seeded in 8-well chamber slides (ibidi catalog no. 80807, 30,000 cells/well) and after 24 hours were treated as described. Cells were fixed using 4% wt/vol formaldehyde (Thermo Fisher Scientific catalog no. PI28906) 10 minutes followed by three washes with DPBS (gibco catalog no. 14190-144), permeabilized with 0.5% wt/vol saponin (MilliporeSigma catalog no. 558255) 15 minutes followed by three wases with DPBS and blocked by incubating in 5% wt/vol BSA (Fisher Scientific catalog no. BP1600) at 37° C for 1 hour. Cells were then incubated with primary antibody mixtures (each at a dilution of 1:100) in 5% wt/vol BSA overnight at 4° C. Following three washes with DPBS cells were incubated with corresponding secondary antibody mixtures (each at a dilution of 1:500) in DPBS 1 hour at room temperature. In the case of Amytracker 680 and Proteostat staining, dye (1:500 vol/vol each) was added and incubated alongside secondary antibodies. Samples were then washed three times with DPBS and further stained with Hoechst 33342 (5 µg/mL, Thermo Fisher Scientific, catalog no. 62249) and in a single case thioflavin T (1.5 mM) for 5 minutes before further washes. Samples were stored in DPBS supplemented with 5 mM $NaN_3$ at 4° C before imaging.

Western blots

Cells were then harvested by trypsinization and cell pellets lysed in 50 µL DPBS containing Roche protease inhibitors (1/10 mL) by sonication (2 X 10 second pulses, 10% amplitude). Protein concentrations were quantified by DC assay, and samples were normalized to 1.5 mg/mL (25 µL) in the lysis buffer following addition of 8.7 µL 4x LDS loading buffer.

Lysates were then resolved on a 14% tris-glycine SDS-PAGE gel (165V, 67 min). Proteins were transferred to nitrocellulose membrane (semi-wet transfer, 25V, 7 min) following blocking with 5% milk/TBST for 1 h at room temperature. Membranes were then incubated with primary antibody (1:1,000 in 5% milk/TBST) overnight at 4 °C before being washed 3x with TBST and incubated with secondary antibody (1:2,000 in 5% milk/TBST) at room temperature for 1 h. The blots were washed 3x and imaged using the Thermo SuperSignal Pico kit.

Docking in Maestro Elements software CovDock:

We docked dipeptide methyl esters using the manufacture recommended standard procedure in Maestros Elements (2024-4 release) CovDock software after minimising the input conformation in ChemDraw pro. A custom docking script was explored but as shown below (Fig. S8) made little difference to the structure which is dominated by the formation of a salt bride between the protonated dipeptide N terminus and the negatively charged Asp 1 residue in the binding site.

Light microscopy and analysis

Stained slides were imaged on a Zeiss LSM 880 laser-scanning confocal microscope with Zen (Black) 2011 SP7 using a Plan-Apo 63x NA 1.4 oil immersion objective, with a pinhole of 90 microns. Hoechst; Ex: 405 nm 1.2%, Em: 410-587 nm, Gain 550. Alexa Fluor 488; Ex: 488 nm 2.0%, pinhole 52 microns, Em: 545-697 nm, Gain 685. Alexa Fluor 555; Ex 514 nm 2.0%, Em: 545-697 nm, Gain 800. AmyTracker 680; Ex: 561 nm 2.0%, pinhole 50 microns, Em: 578-696nm, Gain 625. Alexa Fluor 647; Ex: 633 nm 2.0%, Em: 638-755 nm, Gain 800. Line average 4x with pixel dwell time of 0.55 microseconds.

High-content imaging was performed on a Zeiss Cell Discoverer 7 in confocal mode using a 50x NA 1.2 water immersion objective with a 0.5x tube lens. Hoechst; Ex: 405 nm 3.5%, pinhole 50 microns, Em: 400-595nm, Gain 700. Alexa Fluor 488; Ex: 488 nm 0.6%, pinhole 46 microns, Em: 410-599 nm, Gain 532. Alexa Fluor 555; Ex 514 nm 1.8%, pinhole 39 microns, Em: 400-590, Gain 666. AmyTracker 680; Ex: 561nm 3.0%, pinhole 53 microns, Em: 565-700 nm, Gain 850. Cy5/Alexa Fluor 647; Ex: 633nm 4.0%, pinhole 22 microns, Em: 656-700nm, Gain 625. Line average 4x with a pixel dwell time of 0.42 microseconds.

High-content image analysis was carried out using Arivis Pro ver. 4.2.0 (Carl Zeiss Microscopy GmbH). Preparation of representative images for publication was performed in ImageJ (*54*) (National Institutes of Health).

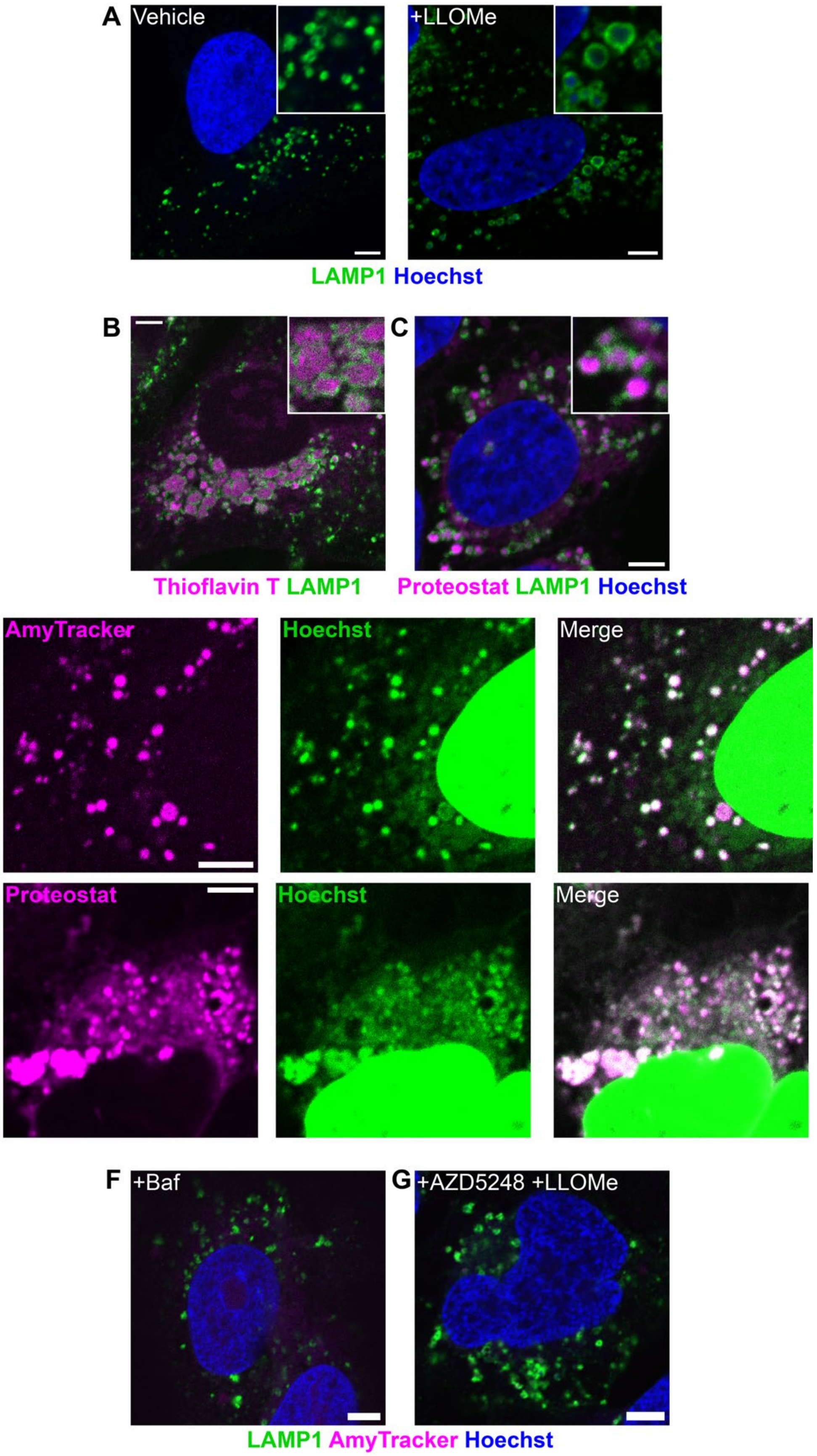
A
Vehicle
+LLOMe
LAMP1 Hoechst
B
C
Thioflavin T LAMP1
Proteostat LAMP1 Hoechst
D
AmyTracker
Hoechst
Merge
E
Proteostat
Hoechst
Merge
F
+Baf
G
+AZD5248 +LLOMe
LAMP1 AmyTracker Hoechst

**Fig. S1. Endolysosomal expansion, thioflavin T, Proteostat, and Hoechst staining upon LLOMe exposure, and pharmacologic nullification of the LLOMe phenotype.**
(**A**) Representative images of U-2 OS cell staining of LAMP1 under basal conditions and upon treatment with LLOMe (1 mM, 10 minutes). (**B-C**) Representative images of U-2 OS cells treated with LLOMe (1 mM, 10 minutes) and stained against LAMP1 and (**B**) Thioflavin T (1 mM), or (**C**) Proteostat (2 µg/mL). (**D-E**)Representative images of Hoechst 33442 and (**D**) AmyTracker or (**E**) Proteostat staining in U-2 OS cells after LLOMe exposure (1 mM, 10 minutes), Hoechst signal intensity artificially increased for clarity. (**F-G**) Representative images of LAMP1 and AmyTracker staining following (**F**) bafilomycin A1 treatment (250 nM, 180 minutes) or (**E**) CTSC inhibitor AZD5248 pretreatment (10 µM, 90 minutes) before LLOMe treatment (1 mM, 60 minutes). All scalebars=5 µm.

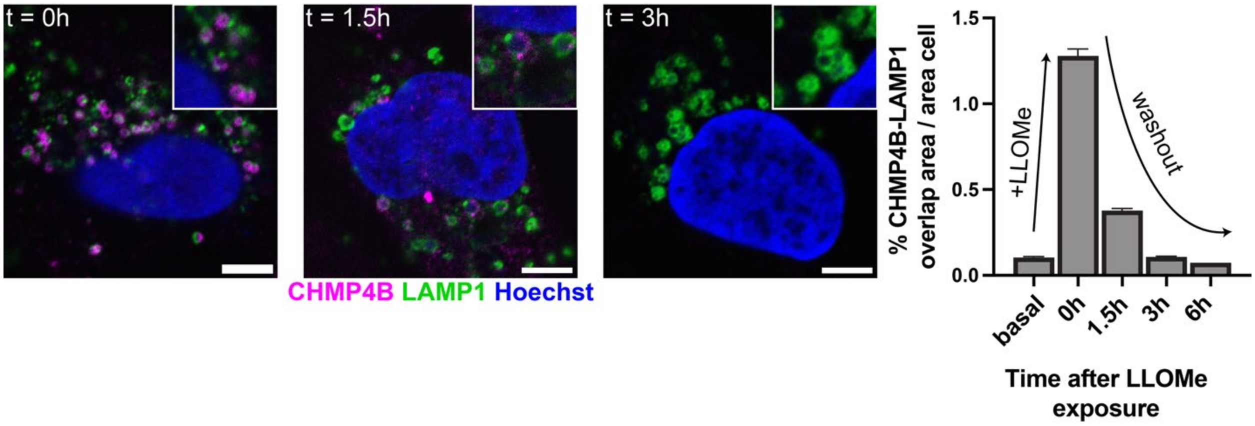


**Fig. S2. LLOMe-induced accumulation and dissipation of CHMP4B on endolysosomal vesicles**.
(Left) representative images in U-2 OS cells of CHMP4B accumulation on LAMP1-positive vesicles at 0, 1.5, and 3-hours after LLOMe (1 mM, 10 minutes) washout. (Right) quantitation of CHMP4B-LAMP1 overlap at the indicated timepoints, n≥740 cells per timepoint. All scalebars=5 µm.

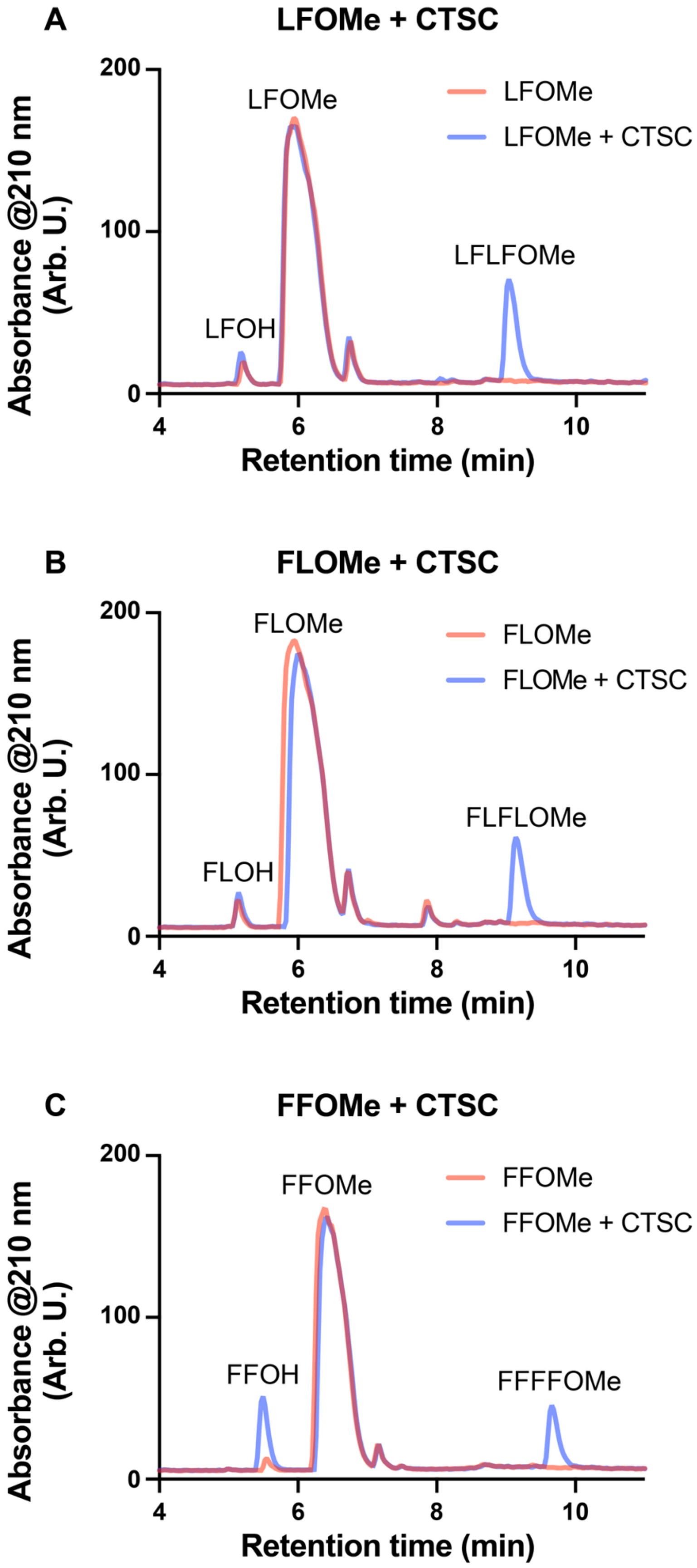
A
LFOMe + CTSC
LFOMe
LFOMe + CTSC
LFOMe
LFLFOMe
LFOH
Absorbance @210 nm (Arb. U.)
Retention time (min)
B
FLOMe + CTSC
FLOMe
FLOMe + CTSC
FLOMe
FLFLOMe
FLOH
Absorbance @210 nm (Arb. U.)
Retention time (min)
C
FFOMe + CTSC
FFOMe
FFOMe + CTSC
FFOMe
FFOH
FFFFOMe
Absorbance @210 nm (Arb. U.)
Retention time (min)

**Fig. S3. *In vitro* reactions among LFOMe, FLOMe, FFOMe, and CTSC.**
LC-MS chromatograms of (**A**) LFOMe alone or LFOMe + CTSC, (**B**) FLOMe alone or FLOMe + CTSC, or (**C**) FFOMe alone or FFOMe + CTSC (20 mM dipeptide and 100 nM CTSC respectively, incubated 8 hours at 37 °C in 20 mM $NaP_i$ pH 6.5 150 mM NaCl). Labeled peaks were identified by mass.

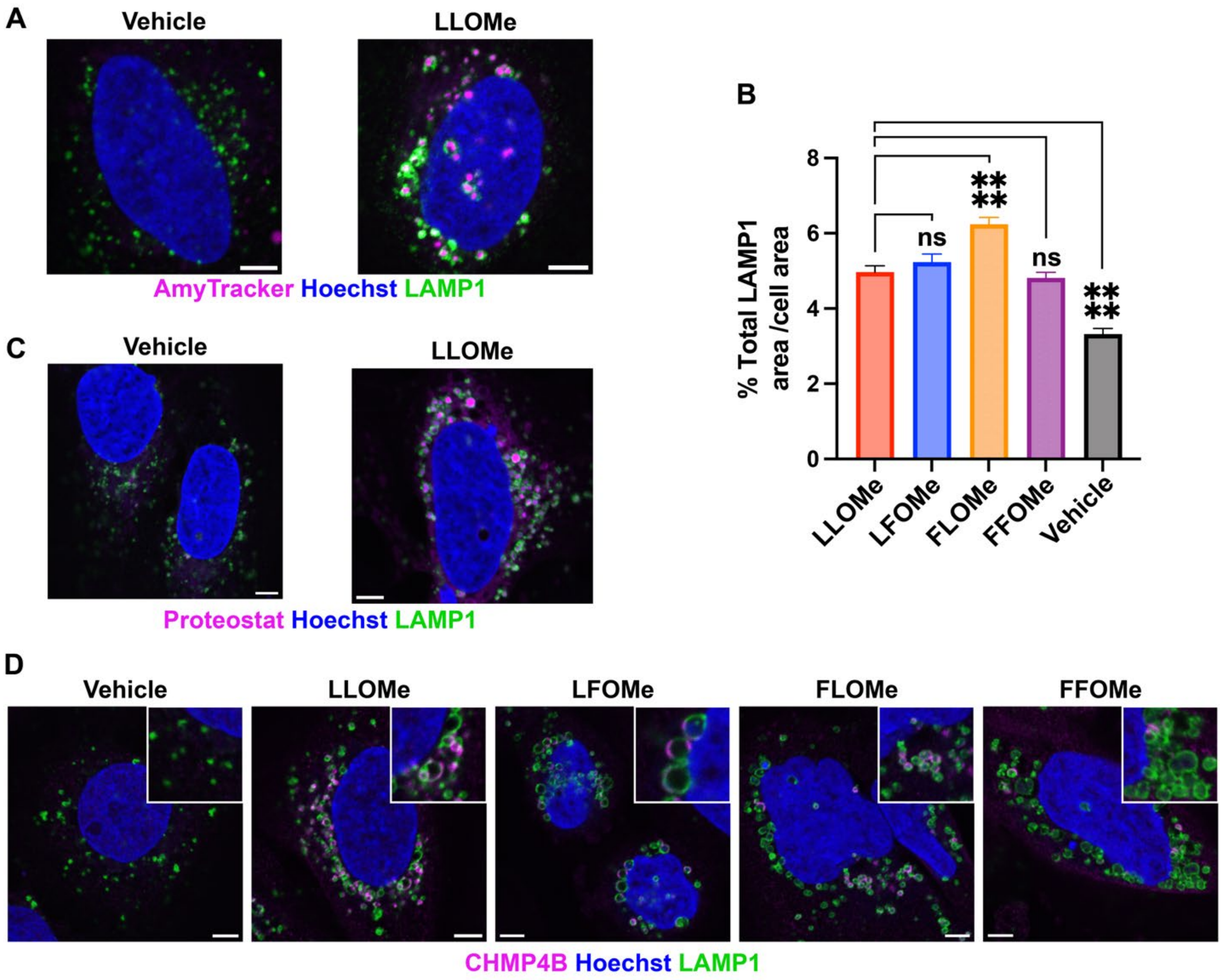


**Fig. S4. Endolysosomal expansion and CHMP4B endolysosomal recruitment in LLOMe, LFOMe, FLOMe, and FFOMe treated cells**

(**A**) Representative images of AmyTracker staining in untreated and LLOMe treated (1 mM, 10 minutes) U-2OS cells. (**B**) quantification of LAMP1 area/cell area upon treatment of LLOMe, LFOMe, FLOMe, FFOMe, or DMSO ($n \geq 135$ cells per treatment), indicated comparisons analyzed using one-way ANOVA, ****=$P \leq 0.0001$, ns=$P > 0.05$ (**C**) Representative images of Proteostat staining in untreated and LLOMe treated (1 mM, 10 minutes) cells. (**D**) Representative images of CHMP4B endolysosomal recruitment upon treatment with DMSO, LLOMe, LFOMe, FLOMe, and FFOMe. All scalebars=5 μm.

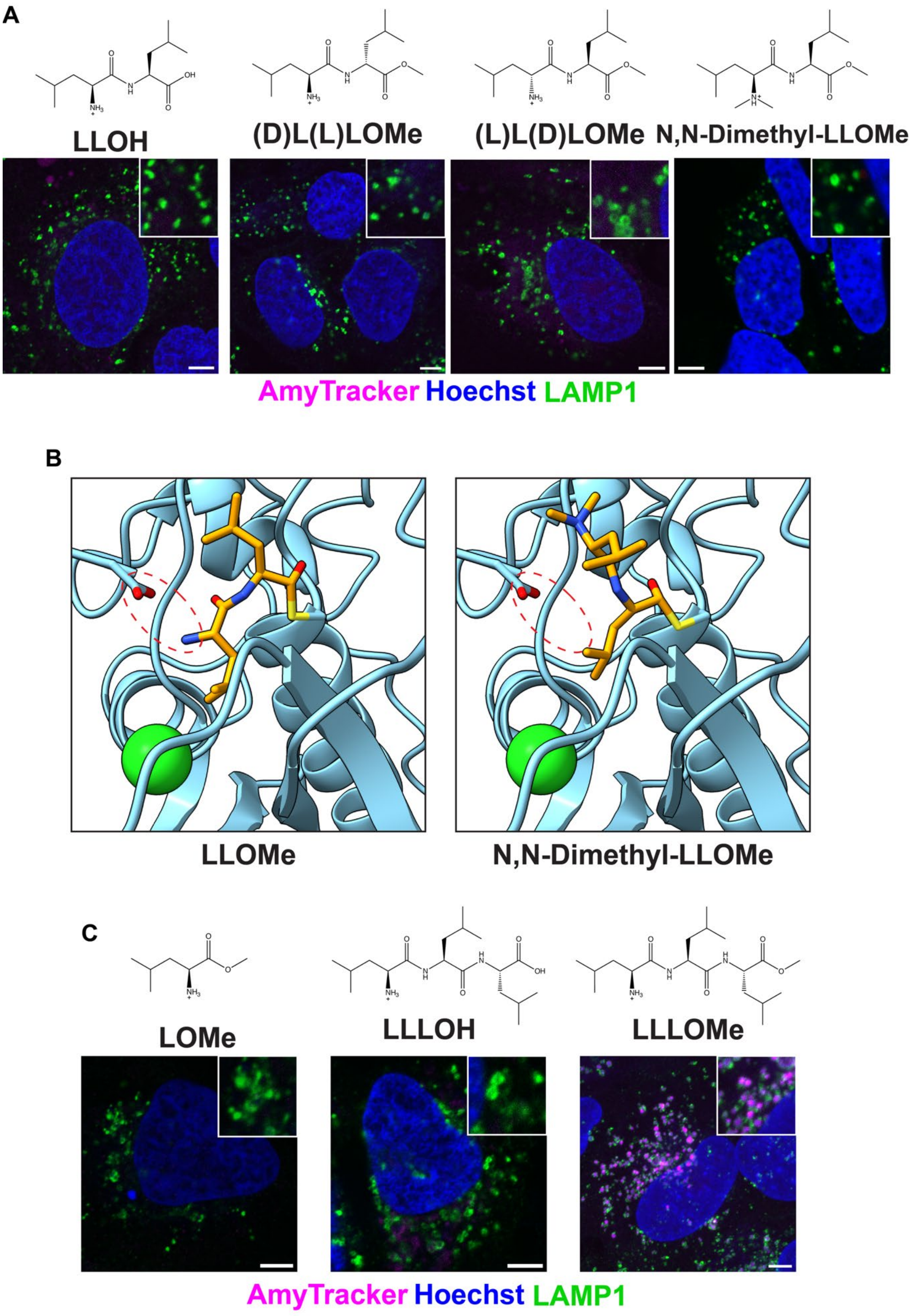
A
LLOH
(D)L(L)LOMe
(L)L(D)LOMe
N,N-Dimethyl-LLOMe
AmyTracker Hoechst LAMP1
B
LLOMe
N,N-Dimethyl-LLOMe
C
LOMe
LLLOH
LLLOMe
AmyTracker Hoechst LAMP1

**Fig. S5. Elucidating LLOMe structural requirements for CTSC-mediated amyloid formation**

(**A**) Representative images of AmyTracker and LAMP1 staining in U-2 OS cells treated with LLOMe derivatives LLOH, (D)-Leu-(L)-Leu-OMe, (L)-Leu-(D)-Leu-OMe, or N-dimethyl LLOMe (1 mM, 60 minutes; structures shown above). (**B**) Docked poses of CTSC covalently bound to ligands LLOMe (left) and N-dimethyl LLOMe at Cys234; red circle denotes the presence or absence of the Asp1-dipeptide N-terminus interaction. (**C**) Representative images of AmyTracker and LAMP1 staining in U-2 OS cells treated with LOMe, LLLOH, or LLLOMe (1 mM, 60 minutes; structures shown above).

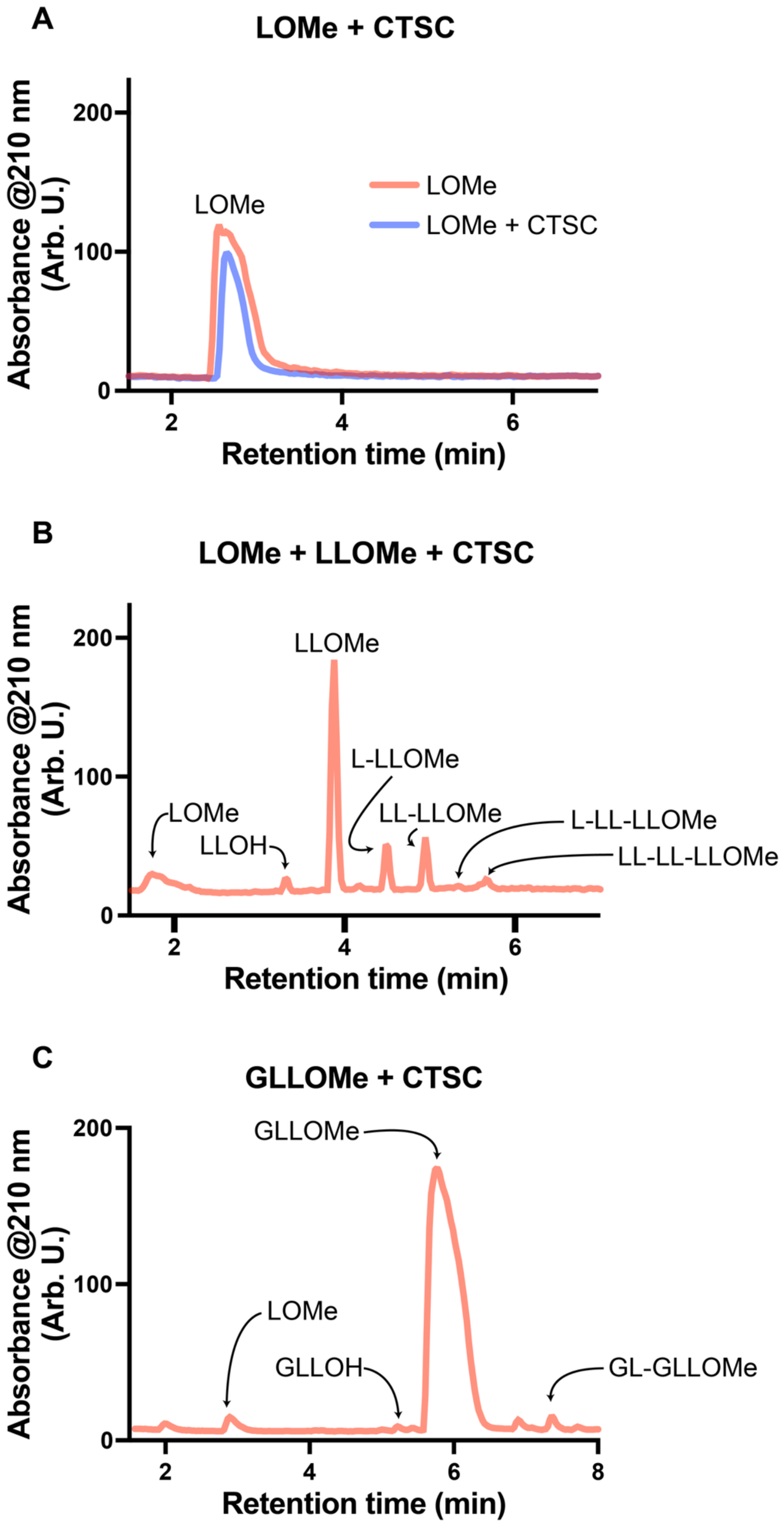
A
LOMe + CTSC
Absorbance @210 nm (Arb. U.)
LOMe
LOMe + CTSC
Retention time (min)
B
LOMe + LLOMe + CTSC
LLOMe
L-LLOMe
LOMe
LLOH
LL-LLOMe
L-LL-LLOMe
LL-LL-LLOMe
C
GLLOMe + CTSC
GLLOMe
LOMe
GLLOH
GL-GLLOMe

**Fig. S6. Elucidating dipeptide methyl ester composition requirements for CTSC-mediated peptide ligation.**

LC-MS chromatograms of (**A**) LOMe alone or LOMe + CTSC (20 mM and 100 nM respectively), (**B**) LOMe + LLOMe + CTSC (10 mM, 10 mM, and 100 nM respectively), and (**C**) GLLOMe + CTSC (20 mM and 100 nM respectively) incubated 8 hours at 37 °C in 20 mM $NaP_i$ pH 6.5 150 mM NaCl. Labeled peaks were identified by mass.

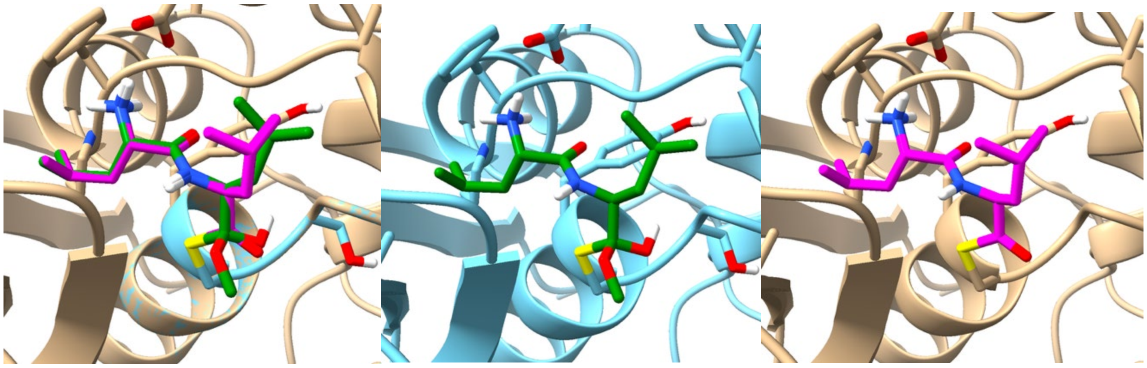

**Fig. S7. Methoxy loss is generally inconsequential in covalent LLOMe-CTSC docking studies.**

(left) Overlay of docking structures of produced starting from LLOMe in Maestro covalent docking function default mode which retains the methoxy group of the dipeptide after Cathepsin C cysteine 234 nucleophilic attack (center, green) vs a custom script which forces the software to produce a thioester and lose the methoxy group (right, magenta). Since the structures overlap almost perfectly in the region of interest (N terminus of dipeptide) we used the default mode for simplicity and manually deleted the methoxy group to avoid confusion in Figure S5.

General procedure for HATU coupling synthesis of di and tripeptide methyl esters:

LLOMe and FFOMe were purchased from Cayman Chemical (catalog no. 16008) and Chem-Impex (catalog no. 12097) respectively and characterized by us using $^{1}H$ NMR. All other dipeptide methyl esters were synthesized on the 100 – 200 mg scale in 40-60% yields using solution phase coupling from their boc and methyl ester starting amino acids as follows:

*Scheme S1 - General reaction scheme for the synthesis of dipeptide methyl esters.*

To a solution of the boc-amino acid (1 equiv.) and amino acid-methyl ester HCl salt (1 equiv.) and HATU (1.05 equiv.) in DMF (0.1 M in amino acid substrate) was added DIEA (2 equiv.) before stirring the reaction at rt. After 5 hours, the solvent was removed under reduced pressure and the mixture redissolved in EtOAc (20 mL). The organic phase was washed with brine (3 × 20 mL) and dried over $Na_2SO_4$ before the solvent was removed under reduced pressure. The crude boc-dipeptide methyl ester was purified using flash column chromatography (Teledyne Isco CombiFlash Rf+ system, 4 g $SiO_2$, Hexane -EtOAC gradient elution, 10-80% over 14 min.) with products typically eluting first around 40% EtOAc. The purity of this boc protected intermediate was verified by $^{1}H$ NMR before boc cleavage in TFA (1 mL) for 5 min at rt. Unbound TFA was completely removed by repeat co–evaporation with MeOH until the mass remained unchanged (which we verified as sufficient evidence of TFA removal using $^{19}F$ NMR on a representative sample). A small portion was taken for $^{1}H$, $^{13}C$ and high-resolution mass spec characterization.

LLOH and LLLOH were synthesized by solid-phase peptide synthesis (SPPS) on Fmoc-Leu-Wang resin under HATU/DIEA coupling conditions. The peptide was cleaved using a mixture of 95/2.5/2.5% TFA/TIPS/$H_2O$ and precipitated with diethyl ether. The crude product was then purified by reverse-phase HPLC and lyophilized overnight. Both LLLOH and LLOH had insufficient solubility in DMSO and $CD_3OD$ on the $^{13}C$ NMR time scale to obtain $^{13}C$ NMR.

Solid peptide preparations were stored at -80 °C until used to make up stock solution in DMSO (1 M).

We obtained high resolution masses for all synthesized compounds on an Agilent 6230 ESI-TOF mass spectrometer coupled to an Agilent 1260 liquid chromatography system. All NMR data was obtained on a Bruker Avance Neo 500 MHz spectrometer with a 5mm BBO probe with a Z-axis gradient.

**Characterizations of synthesized dipeptides:**

**LFOMe**

**$^{1}$H NMR (500 MHz, $CD_3OD$):**
7.29 (2H, m), 7.23 (3H, m), 4.70 (1H, m), 3.86 (1H, m), 3.68 (3H, s), 3.19 (1H, dd), 3.04 (1H, m), 1.74-1.62 (3H, m), 0.99 (3H, d), 0.96 (3H, d).

**$^{13}$C NMR (500 MHz, $CD_3OD$):**
173.0, 170.9, 138.0, 130.1, 129.6, 128.0, 55.6, 52.8, 52.7, 41.7, 38.0, 25.2, 23.1, 21.9.

**HRMS-ESI:** *m/z 293.1837* found, 293.1865 *calculated for [M+H]* $C_{16}H_{25}N_2O_3$.

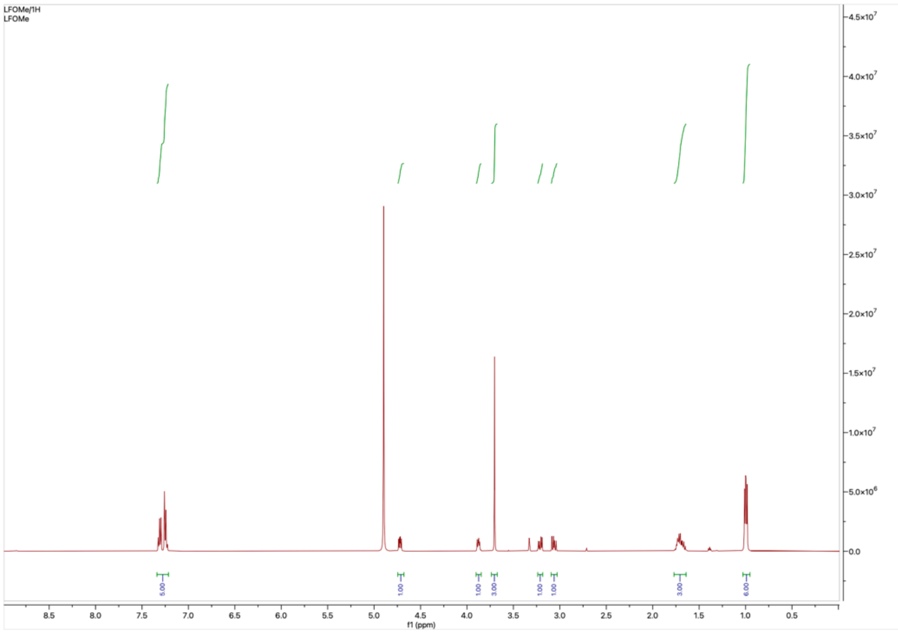


**Figure S8 - $^{1}$H NMR spectra of LFOMe in $CD_3OD$.**

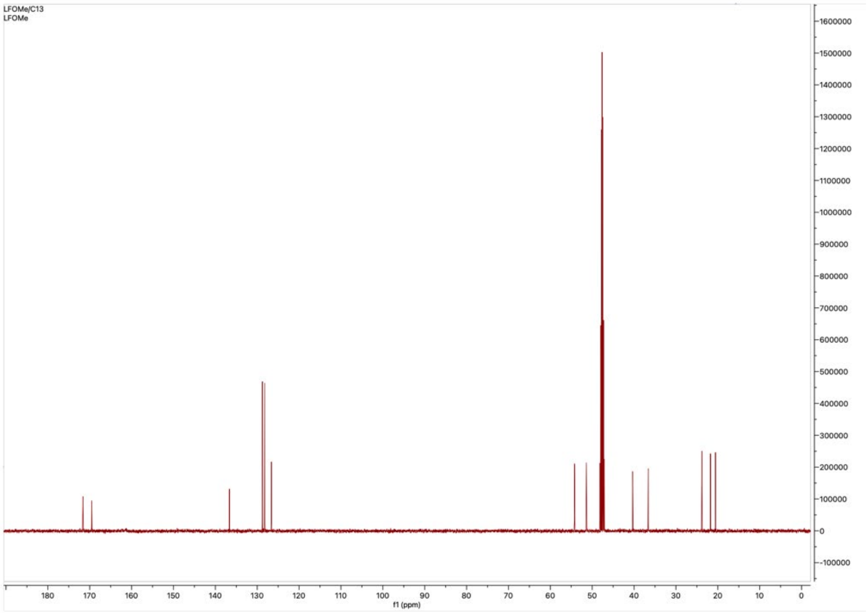


**Figure S9 -$^{13}$C NMR spectra of LFOMe in $CD_3OD$.**

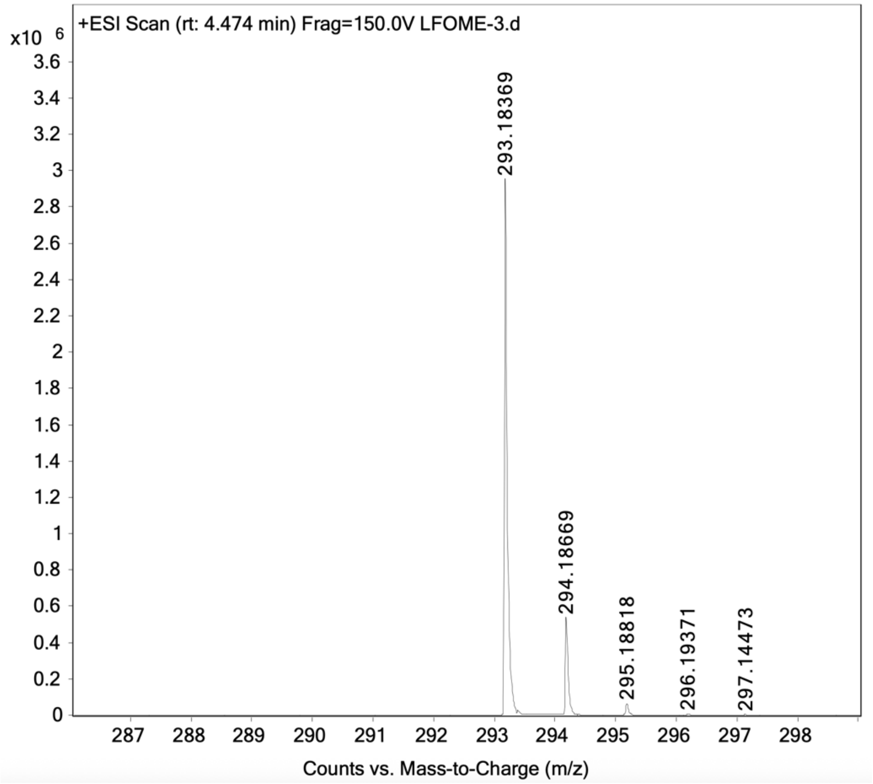


**Figure S10 - $[M+H]^+$ ion found using HRMS on LFOMe.**

**FLOMe**

**$^{1}$H NMR (500 MHz, $CD_3OD$):**

7.33 (5H, m), 4.50 (1H, t), 4.16 (1H, t), 3.70 (3H, s), 3.27 (1H, d), 3.03 (1H, m), 1.59-1.73 (3H, m), 0.96 (3H, d), 0.92 (3H, d).

**$^{13}$C NMR (500 MHz, $CD_3OD$):**

173.9, 169.8, 135.5, 130.6, 130.1, 128.8, 55.5, 52.8, 52.3, 41.8, 38.5, 25.8, 23.2, 21.8.

**HRMS-ESI:** *m/z 293.1893* found*,* 293.1865 *calculated for [M+H]* $C_{16}H_{25}N_2O_3$.

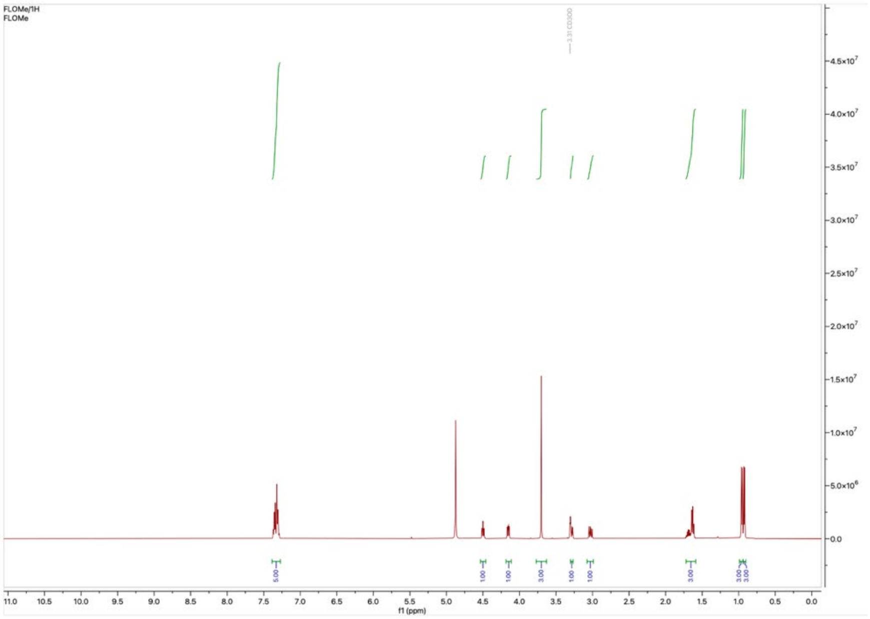

**Figure 11 - $^{1}$H NMR spectra of FLOMe in $CD_3OD$.**

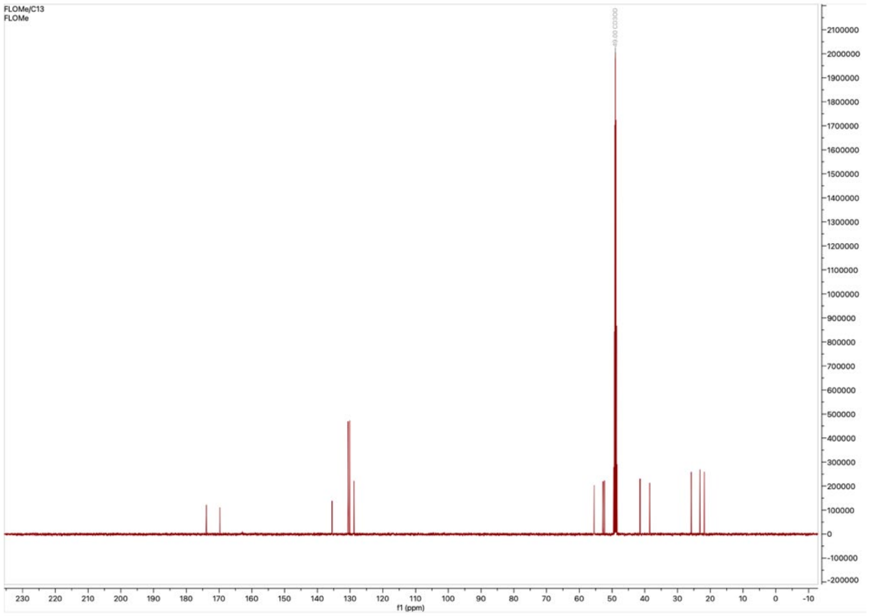

**Figure 12 - $^{13}$C NMR spectra of FLOMe in $CD_3OD$.**

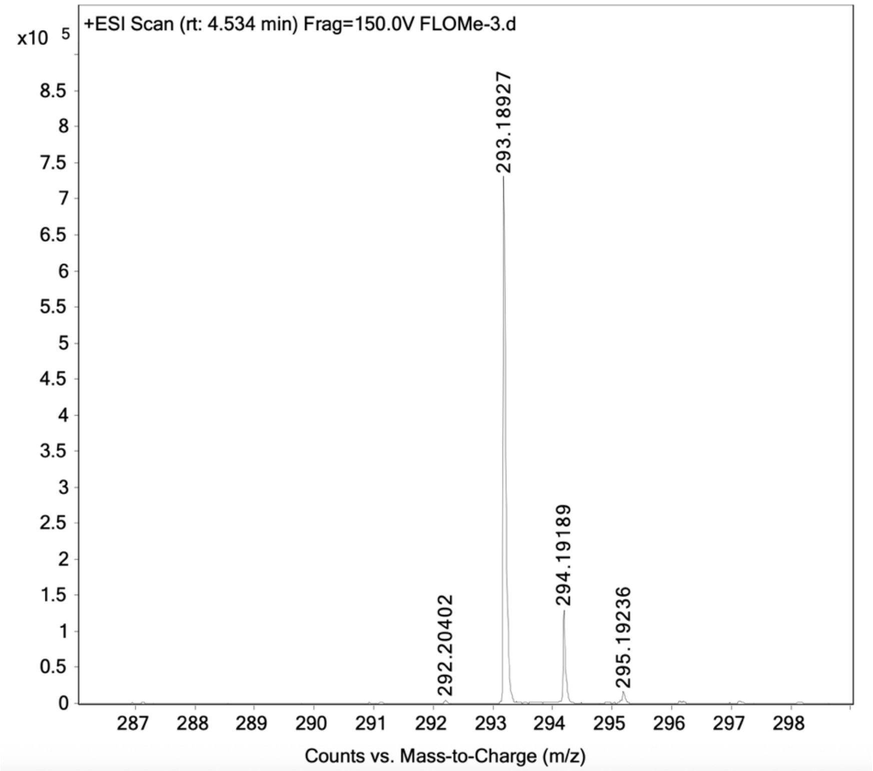


**Figure S13 - $[M+H]^+$ ion found using HRMS on FLOMe.**

**(*D*)LLOMe**

**$^{1}$H NMR (500 MHz, $CD_3OD$):** 4.50 (t, 1H), 3.89 (m, 1H), 3.71 (s, 3H), 1.80-1.62 (m, 6H), 1.05-0.92 (m, 12H).

**$^{13}$C NMR (500 MHz, $CD_3OD$):** 174.1, 170.9, 52.8, 52.7, 52.3, 41.8, 41.2, 25.9, 25.2, 23.2, 23.1, 22.0, 21.7.

**HRMS-ESI:** *m/z 259.2033 found*, *259.2022 calculated for [M+H]* $C_{13}H_{27}N_2O_3$.

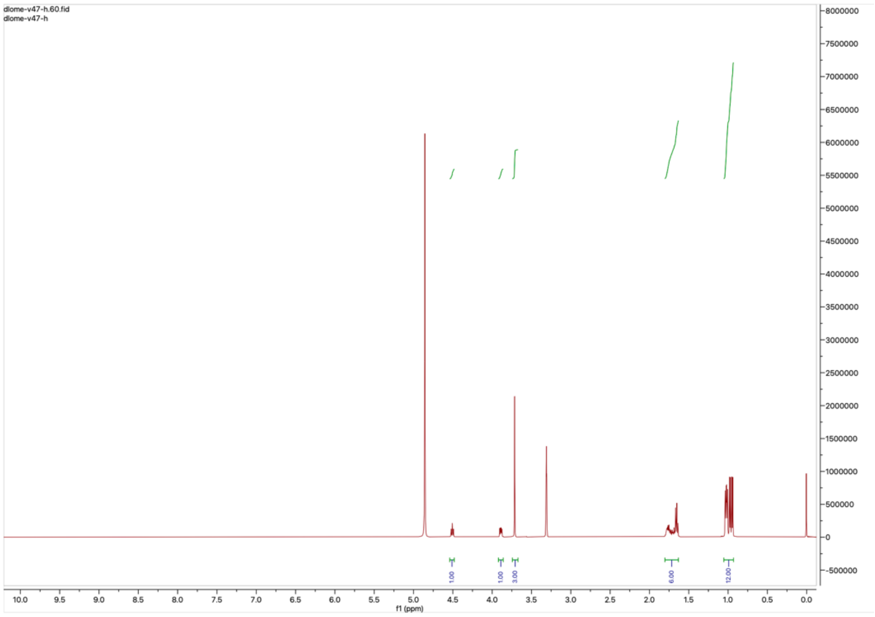

**Figure S14 - $^{1}$H NMR spectra of (D)LLOMe in $CD_3OD$.**

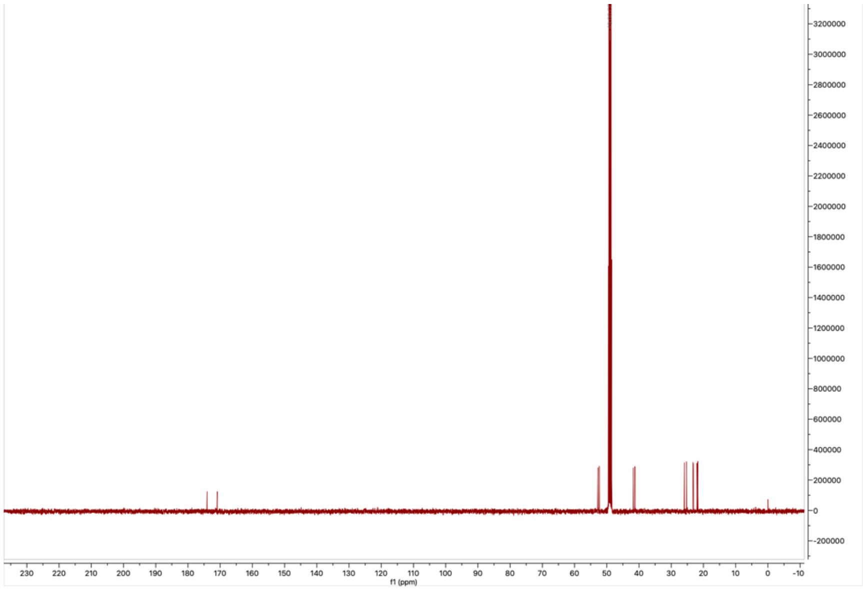


**Figure S15 - $^{13}$C NMR spectra of (D)LLOMe in $CD_3OD$.**

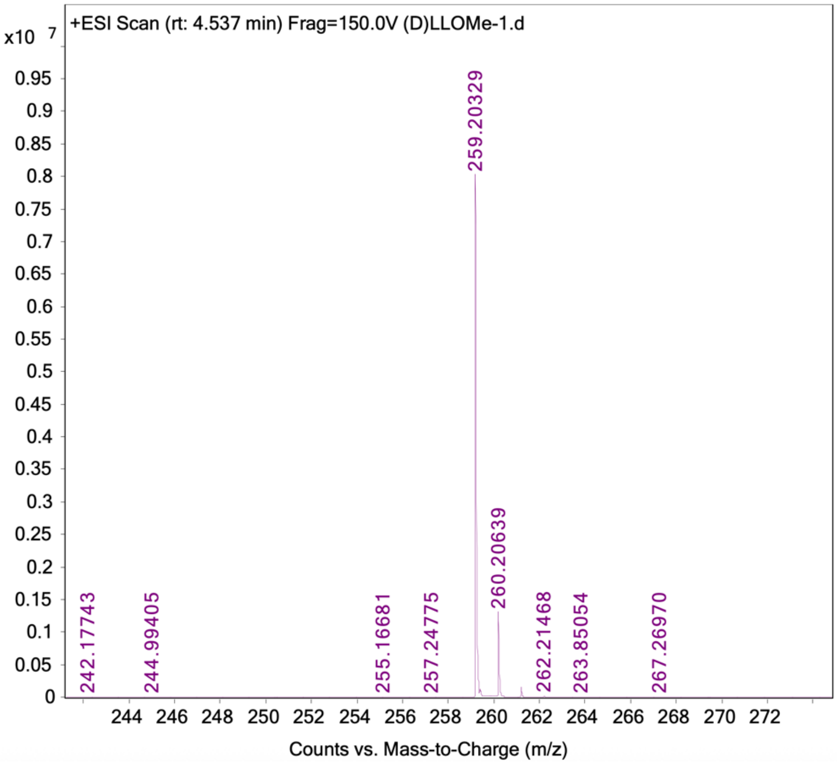


**Figure S16 - $[M+H]^+$ ion found using HRMS on (D)LLOMe.**

**LLLOMe**

**$^1$H NMR (500 MHz, $CD_3OD$):** 4.51 (t, 1H), 4.46 (m, 1H), 3.88 (m, 1H), 3.72 (s, 3H),1.77-1.53 (m, 9H), 1.01-0.88 (m, 18H).

**$^{13}$C NMR (500 MHz, $CD_3OD$):** 174.4, 174.2,170.5, 53.1, 52.8, 52.6, 41.9, 41.8, 41.3, 25.9, 25.8, 25.3, 23.4, 23.3, 23.1, 22.3, 22.1, 21.7.

**HRMS-ESI:** *m/z 372.2864* found*, 372.2862 calculated for [M+H]* $C_{19}H_{37}N_3O_4$.

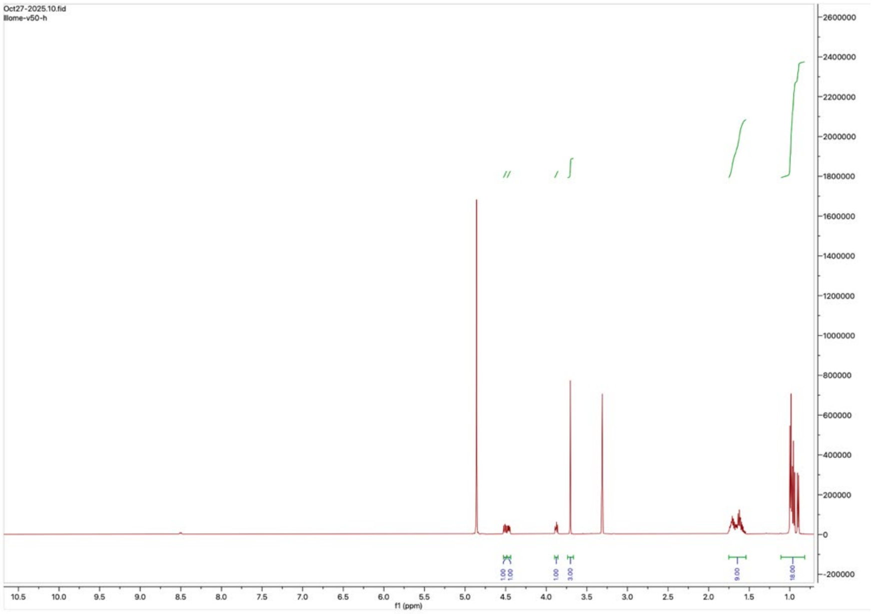

**Figure S17 -$^1$H NMR spectra of LLLOMe in $CD_3OD$.**

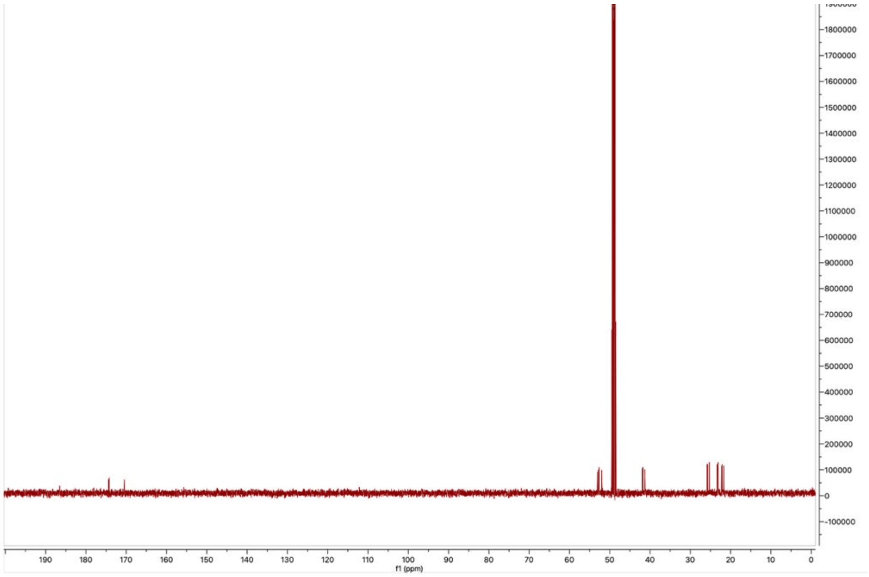

**Figure S18 - $^{13}C$ NMR spectra of LLLOMe in $CD_3OD$.**

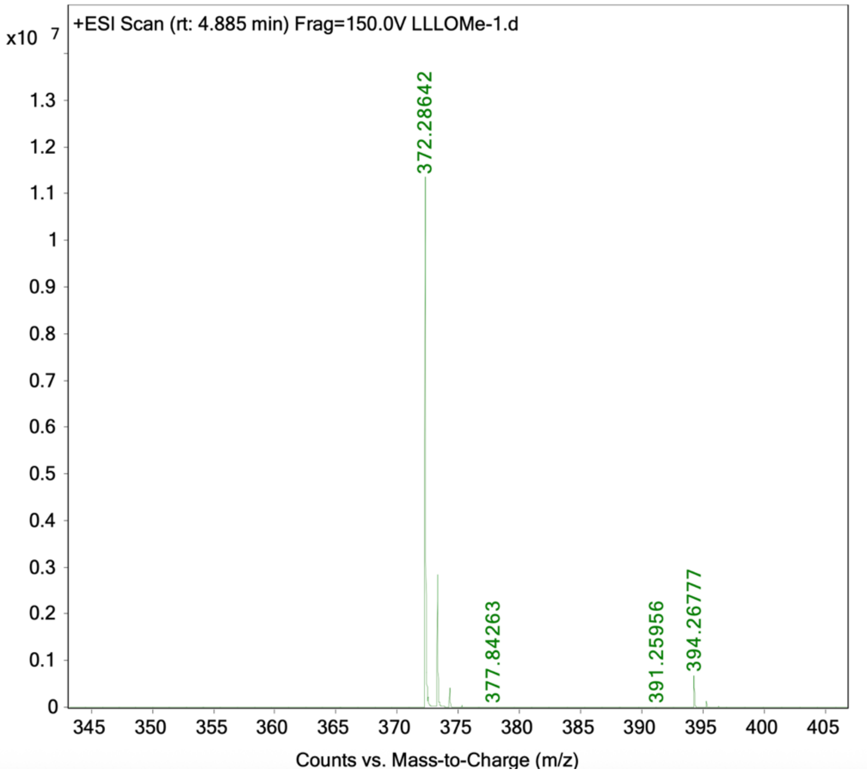


**Figure S19 - $[M+H]^+$ ion found using HRMS on LLLOMe.**

**GLLOMe**

**$^{1}$H NMR (500 MHz, $CD_3OD$):** 8.52 (d,1H), 4.50-4.42 (m, 2H), 3.70 (s, 3H), 3.69 (s, 2H), 1.76-1.66 (m, 2H), 1.65-1.55 (m, 4H), 1.00-0.89 (m, 12H).

**$^{13}$C NMR (500 MHz, $CD_3OD$):** 174.8, 174.5, 167.1, 53.1, 52.7, 52.2, 42.2, 41.4, 41.3, 25.9, 25.8, 23.4, 23.3, 22.0, 21.8.

**HRMS-ESI:** *m/z 316.2240 found,* 316.2236 *calculated for [M+H]* $C_{15}H_{30}N_3O_4$.

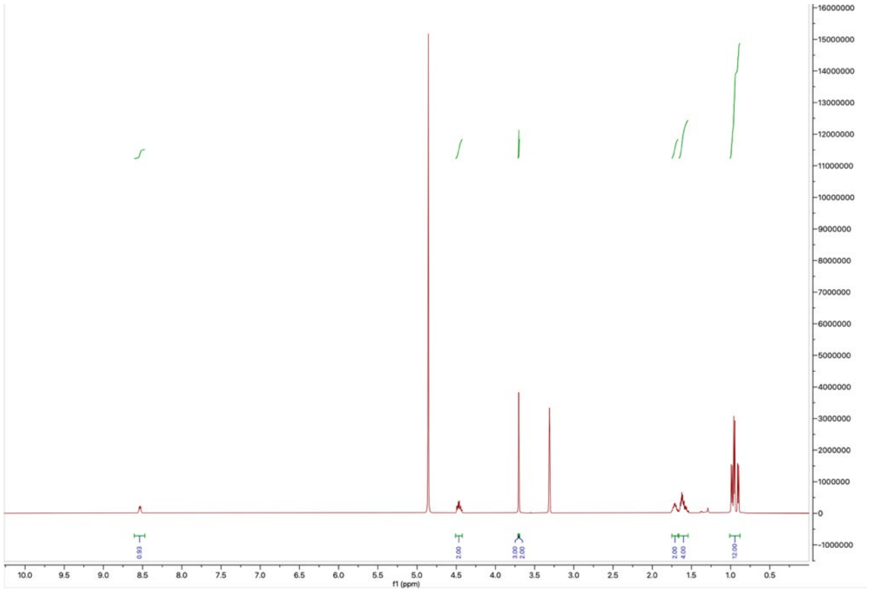

**Figure S20 - $^{1}$H NMR spectra of GLLOMe in $CD_3OD$.**

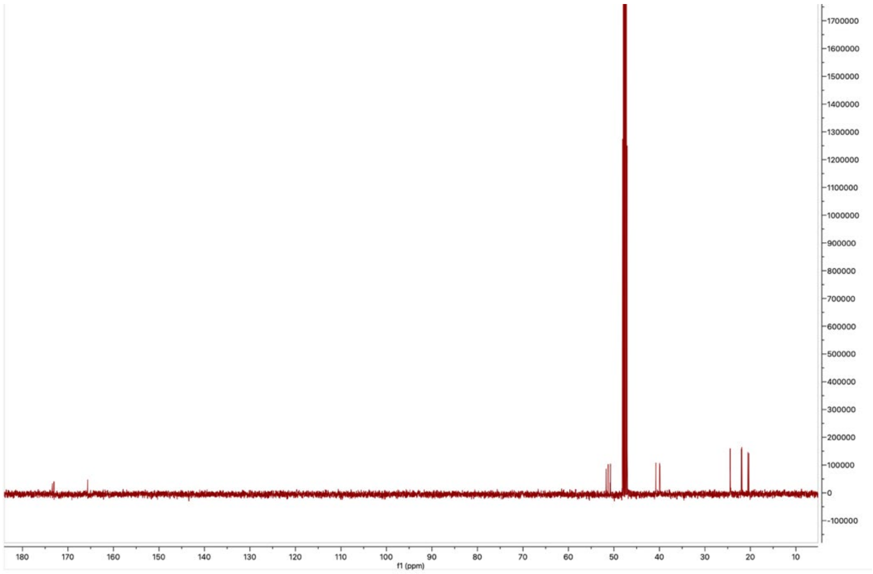

**Figure S21 - $^{13}$C NMR spectra of GLLOMe in $CD_3OD$.**

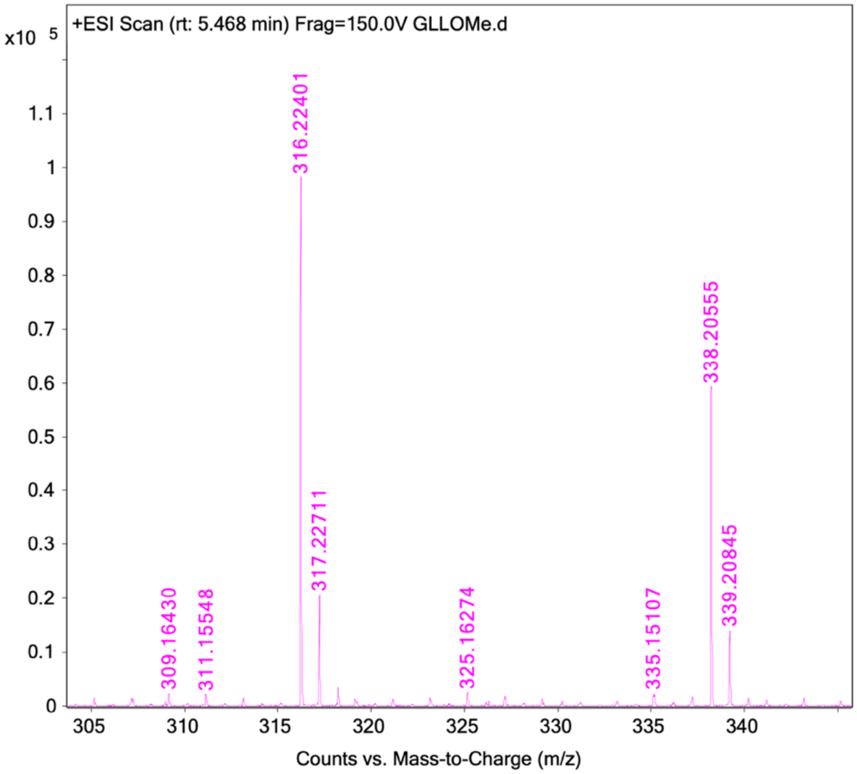


**Figure S22 - $[M+H]^+$ ion found using HRMS on GLLOMe.**

**LLLOH**

**$^1$H NMR (500 MHz, $CD_3OD$):** 4.57 (s,1H), 4.47 (m, 1H), 4.33 (m, 1H), 3.81 (m, 1H), 1.76 – 1.52 (m, 9H), 1.03-0.86 (m, 18H).

**HRMS-ESI:** *m/z 358.2722 found,* 358.2705 *calculated for [M+H]* $C_{18}H_{36}N_3O_4$.

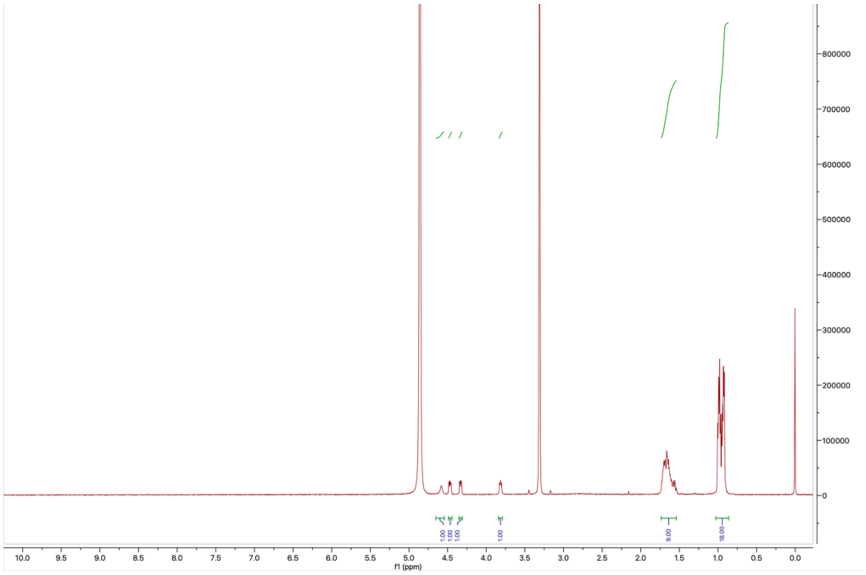


**Figure S23 - $^1$H NMR spectra of LLLOH in $CD_3OD$.**

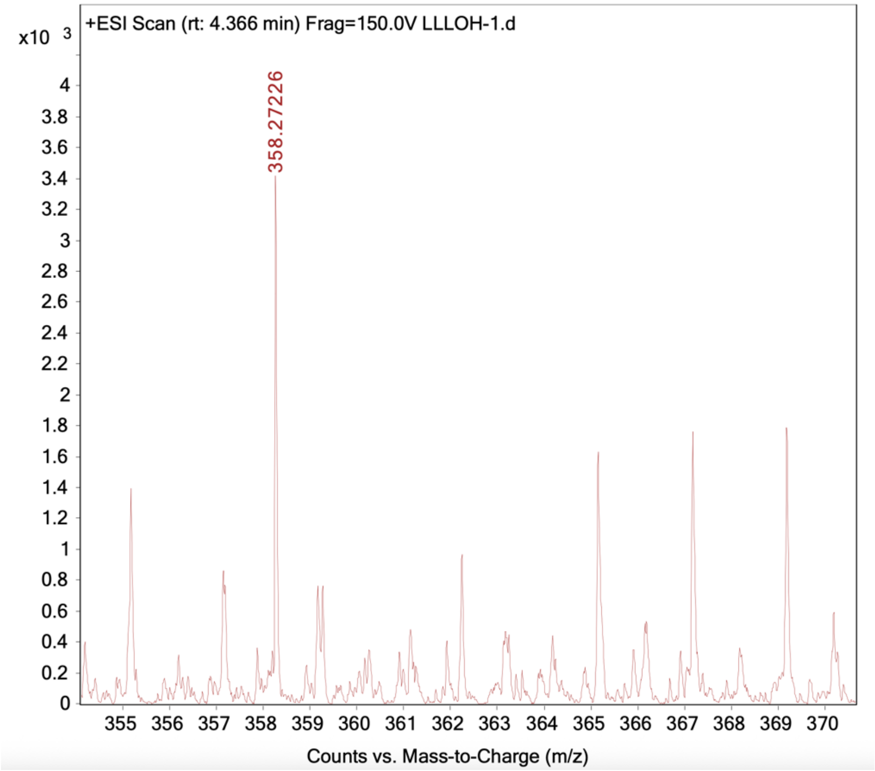


**Figure S24 - $[M+H]^+$ ion found using HRMS on LLLOH.**

**LLOH**

**$^1$H NMR (500 MHz, $CD_3OD$):** 4.57 (s,1H), 4.47 (m, 1H), 4.33 (m, 1H), 3.81 (m, 1H), 1.76 – 1.52 (m, 9H), 1.03-0.86 (m, 18H).

**HRMS-ESI:** *m/z 245.1869 found,* 245.1865 *calculated for [M+H]* $C_{12}H_{25}N_2O_3$.

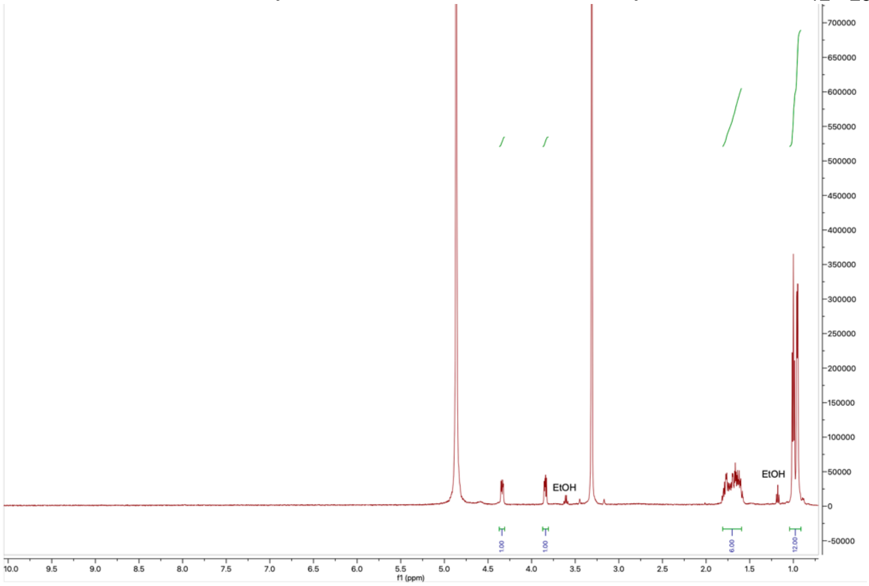


**Figure S25 -$^1$H NMR spectra of LLOH in $CD_3OD$.**

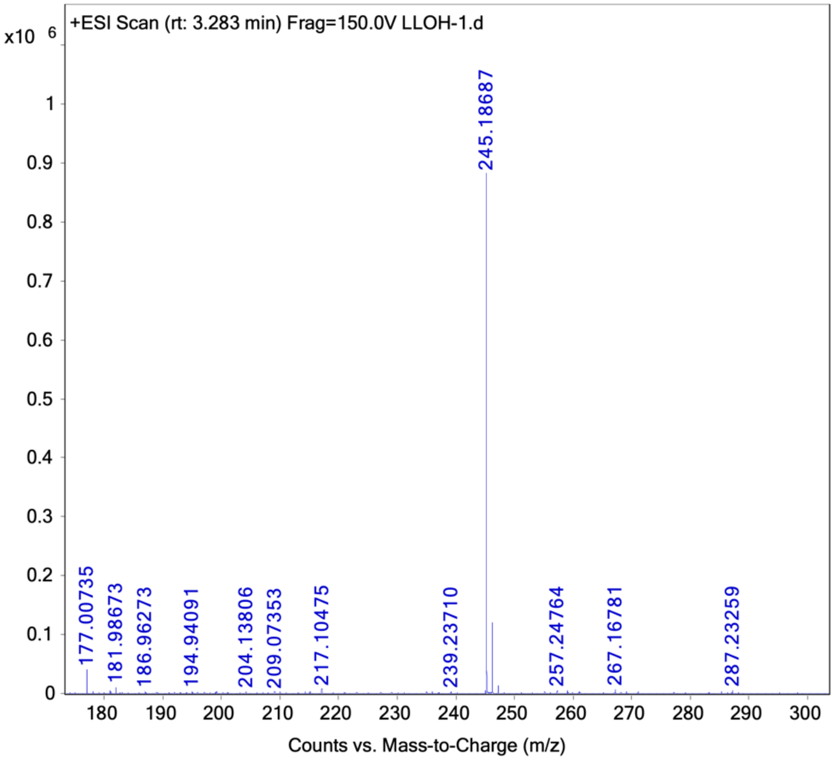


**Figure S26 - [M+H]+ ion found using HRMS on LLOH.**

**Movies S1-S3. Cryo-electron tomogram series of lysosomes isolated from HEK293T cells treated with LLOMe.**

Tilt-series movies of three lysosomes highlighted in **Fig. 2** (pixel size = 1.51 Å, scalebar=50 Å, series spans a range of –45° to +45° in 3° increments).
